# Lipidomic scanning of self-lipids identifies headless antigens for natural killer T cells

**DOI:** 10.1073/pnas.2321686121

**Published:** 2024-08-14

**Authors:** Tan-Yun Cheng, T. Praveena, Srinath Govindarajan, Catarina F. Almeida, Daniel G. Pellicci, Wellington C. Arkins, Ildiko Van Rhijn, Koen Venken, Dirk Elewaut, Dale I. Godfrey, Jamie Rossjohn, D. Branch Moody

**Affiliations:** ^a^Division of Rheumatology, Inflammation, and Immunity, Brigham and Women’s Hospital and Harvard Medical School, Boston, MA 02210; ^b^Infection and Immunity Program and Department of Biochemistry and Molecular Biology, Biomedicine Discovery Institute, Monash University, Clayton, VIC 3800, Australia; ^c^Molecular Immunology and Inflammation Unit, Vlaams Instituut voor Biotechnologie, Center for Inflammation Research, Ghent University, 9052 Ghent, Belgium; ^d^Faculty of Medicine and Health Sciences, Department of Internal Medicine and Pediatrics (Rheumatology unit), Ghent University, 9000 Ghent, Belgium; ^e^Department of Microbiology & Immunology, Peter Doherty Institute for Infection and Immunity, University of Melbourne, Melbourne, VIC 3010, Australia; ^f^Institute of Infection and Immunity, Cardiff University School of Medicine, Heath Park, Cardiff CF14 4XN, UK

**Keywords:** CD1d, NKT cells, self antigens, lipids

## Abstract

To broadly measure the spectrum of cellular self-antigens for natural killer T cells (NKT), we developed a sensitive lipidomics system to analyze lipids trapped between CD1d and NKT T cell receptors (TCRs). We captured diverse antigen complexes formed in cells from natural endogenous lipids, with or without inducing endoplasmic reticulum (ER) stress. After separating protein complexes with no, low, or high CD1d–TCR interaction, we eluted lipids to establish the spectrum of self-lipids that facilitate this interaction. Although this unbiased approach identified fifteen molecules, they clustered into only two related groups: previously known phospholipid antigens and unexpected neutral lipid antigens. Mass spectrometry studies identified the neutral lipids as ceramides, deoxyceramides, and diacylglycerols, which can be considered headless lipids because they lack polar headgroups that usually form the TCR epitope. The crystal structure of the TCR–ceramide–CD1d complex showed how the missing headgroup allowed the TCR to predominantly contact CD1d, supporting a model of CD1d autoreactivity. Ceramide and related headless antigens mediated physiological TCR binding affinity, weak NKT cell responses, and tetramer binding to polyclonal human and mouse NKT cells. Ceramide and sphingomyelin are oppositely regulated components of the “sphingomyelin cycle” that are altered during apoptosis, transformation, and ER stress. Thus, the unique molecular link of ceramide to NKT cell response, along with the recent identification of sphingomyelin blockers of NKT cell activation, provide two mutually reinforcing links for NKT cell response to sterile cellular stress conditions.

## Significance

Natural killer T (NKT) cells are an abundant T cell subset that recognizes CD1d and lipid antigens. Whereas most antigens are glycolipids from bacteria, NKT cells are selected, reside in tissues, and are activated in sterile conditions. Therefore, we comprehensively profiled human and mouse cells for self-antigens, identifying ceramides as an abundant and highly regulated class of self-antigens. These data conclusively identify a molecular target of NKT cells in the sphingomyelin cycle, which is regulated in cell stress and transformation. Further structural data disrupt the corecognition model, where NKT cells normally recognize protruding glycolipid headgroups that function as epitopes. Here, ceramide is a headless molecule, and a ternary crystal structure shows contact of the NKT cell receptor with CD1d itself.

As contrasted with the high diversity of T cell receptors (TCRs) that recognize major histocompatibility (MHC) proteins, “invariant” natural killer T (NKT) cells are defined by highly conserved TCRs that recognize CD1d (1). Compared to conventional naive T cells, NKT cells are resident at high numbers in liver and other tissues, where their nearly invariant TCRs allow them to act as a cohort with high effector function and modulate systemic immune responses in models of infection, cancer, and autoimmune disease (2). The identification of α-galactosyl ceramide (αGalCer) antigens from a compound library usefully provided highly potent but non-natural agonists for NKT cell studies (3, 4). However, understanding natural antigens that control NKT response has been a central and unresolved question in the field, especially for self-antigens that might mediate positive selection in the thymus or inflammatory responses in sterile conditions (5, 6).

To identify natural antigens, a common approach was to test NKT response for natural molecules that resemble synthetic αGalCer superagonists. Microbial α-linked antigens have been identified in *Sphingomonas* species (7, 8), the gut symbiont, *Bacteroides fragilis* (9), *Streptococcus pneumonia,* and *Borrelia burgdorferi* (10, 11). Considering self-antigens, α-linked hexosyl lipids have been detected in milk (12) and were suggested to be present in normal tissues based on antibody staining (13). There is also convincing evidence for natural NKT ligands lacking structural relatedness to αGalCer. For example, self-phospholipids, such as phosphatidylinositol (PI) and phosphatidylethanolamine (PE) (14), as well as lysophospholipids (15) and peroxisome-derived ether-linked lipids can activate NKT cells (16). However, phospholipid antigens act with lower potency compared to αGalCer compounds. Similarly, mouse isogloboside 3 activates NKT cells (17), but its expression in humans (18) and its role in NKT cell selection have been questioned (19).

Despite the many candidates, no clear immunodominant self-antigens that mediate inflammation in sterile conditions have been identified. However, two groups recently identified signaling pathways that involve the unfolded protein response and related mechanisms of endoplasmic reticulum (ER) stress that lead to NKT cell response (5, 6). Specifically, stress-inducing agents, thapsigargin or tunicamycin, provoked downstream responses involving protein kinase R-like ER kinase (PERK) and inositol-requiring enzyme 1A (IRE-1a) that activated NKT cells. NKT cell stimulants were recovered from experimentally stressed antigen-presenting cells (APCs) using organic solvents, suggesting that the mechanism involves generation of self-lipids presented by CD1d rather than NKT cell activation by cytokines. However, the stimulatory compounds could not be identified with conventional approaches that rely on activation assays of NKT cell response using exogenously added self-antigens (5, 6).

Here, we implemented a self-antigen discovery approach (12) whereby the non-CD1d-binding lipid biomass is first discarded, and CD1d–self-lipid complexes are separated into those that do or do not bind an autoreactive NKT TCR. Then, lipidomic analysis of all self-compounds in CD1d–TCR complexes could detect any self-lipid that ligates CD1d to NKT TCRs. TCR binding was mediated by ceramide and structurally related molecules that were not widely recognized to be antigens. These molecules share one striking biochemical feature: lack of polar headgroups that normally protrude from CD1 to form TCR epitopes. Structural analysis of CD1d–ceramide–NKT TCR complexes showed that ceramide was buried within CD1d, and most NKT TCR contacts were made with CD1d itself. Tetramer staining also revealed polyclonal populations of human and mouse NKT cells recognizing headless antigens. Although less potent than αGalCer superagonists, these weakly acting ceramide antigens are abundant and highly regulated in cellular stress pathways (20), providing an unexpected molecular explanation for NKT cell activation in the absence of infection.

## Results

### Thapsigargin Induces Lipid Antigens for NKT Cells.

**Fig. 1.**
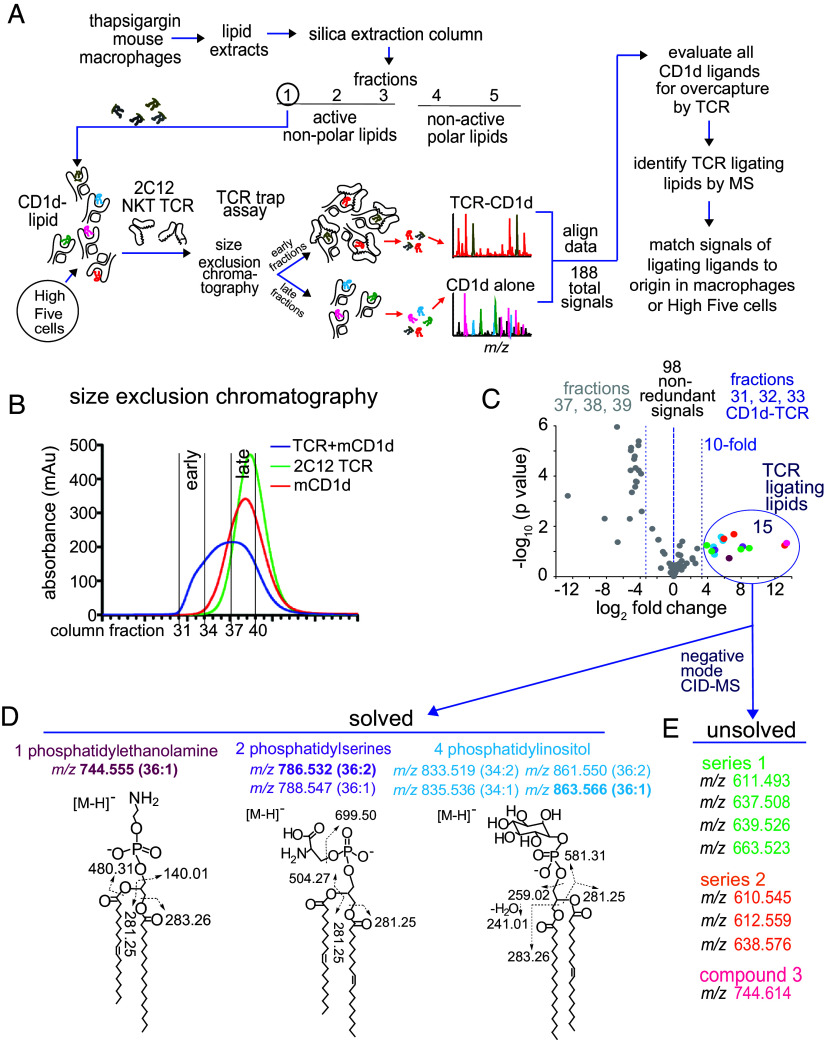
Lipidomic analysis of CD1d–lipid complexes that bind the 2C12 TCR. (*A*) Fraction 1 from low-resolution enrichment of NKT activating factors from mouse J774.2 cells (6) was added to mouse CD1d complexes produced in High Five insect cells, followed by 2C12 NKT TCR complexation, size exclusion chromatography, lipid elution, and lipidomics using HPLC–TOF–MS. (*B*) Mouse CD1d and 2C12 TCR, mixed or added separately, entered into size exclusion chromatography and were subjected to protein monitoring by ultraviolet light and gel electrophoresis (*SI Appendix*, Fig. S2) to determine the CD1d to TCR ratio, and allowing normalization of lipid eluents based on protein abundance. CD1d and TCR fractions 31 to 33 were considered cocomplexed based on their appearance in fractions eluting prior to separately added TCR and CD1d. (*C*) The eluents of protein fractions were detected by reversed-phase HPLC–MS analyzed on an ion trap (*SI Appendix*, Fig. S3) and TOF (Fig. 2) mass spectrometers. Lipidomics analysis compared signals from three early eluting CD1d–TCR complexed fractions (31 to 33) to the three late eluting noncomplexed fractions (37 to 39). Ions were considered enriched in TCR–CD1d complexes when the average intensity was >10-fold higher in the cocomplex fractions compared to late eluting fractions. Color coding denotes lipids of the same class with chain length (CH2) and saturation states (H2). (*D*) Certain features matched the known mass of common phospholipids, which were confirmed by CID-MS in the negative mode with the indicated diagnostic fragments. (*E*) Eight lipids of known mass remained unsolved in negative mode CID-MS, so underwent further analysis in the positive mode as cations.

Using thapsigargin and tunicamycin as inducers of ER stress in mouse bone marrow macrophages or the J774 mouse macrophage line, we previously found that lipids extracted from treated cells activated a mouse hybridoma expressing a type I or invariant mouse NKT TCR, 2C12 (6). Stimulatory molecules from J774 macrophages extracted into organic solvents and activated NKT cells when combined with recombinant plate-bound CD1d. Prior work using silica column chromatography of thapsigargin-treated macrophage lipids showed that the highest 2C12 response was to fraction 1 (Fig. 1 and *SI Appendix*, Fig. S1). Fraction 1 contained hydrophobic neutral lipids that are structurally distinct from known antigens, such as glycolipids and phospholipids that eluted in fractions 4 to 5 (6). These outcomes suggested that ER stress induced previously unknown antigens, but conventional chromatography methods used to identify the lipids proved infeasible. Therefore, we implemented a method to sensitively isolate and detect many lipids trapped between CD1d and TCRs (Fig. 1*A*) (12, 21).

### Trapping Lipids between CD1d and the TCR.

This approach was relatively unbiased because it relied on endogenous cellular lipid pools and natural cellular loading mechanisms (22). First, the soluble and transmembrane truncated mouse CD1d was produced in High Five insect cells. Then secreted CD1d–lipid complexes were mixed with fraction 1 lipid of thapsigargin-treated mouse macrophages, combined with soluble 2C12 TCR heterodimers, and passed over a size exclusion column (Fig. 1 *A* and *B*). Protein elution across fractions 31 to 39 was monitored by ultraviolet light absorbance (Fig. 1*B*) and gel electrophoresis (*SI Appendix*, Fig. S2). Confirming prior work (12), the complexation of CD1d and TCR was demonstrated by their detection in size-excluded fractions (31 to 33) that eluted ahead of uncomplexed CD1d and TCR monomers.

### Detecting Molecules at the CD1d–TCR Interface.

Overall, 8 × 10^7^ cells, containing ~7.8 mg of total cellular lipid, yielded 4.2 mg of secreted CD1 protein, which would be estimated to carry ~75 µg lipid based on 1:1 lipid-CD1d stoichiometry and an average mass of 750 (23). From this total CD1d-bound lipid value, ~20% is captured by early eluting CD1d complexes. Thus, the TCR trap method removes ~99.8% of cellular lipids, that were not bound in CD1d–TCR complexes, prior to initiating detailed high-performance liquid chromatography–mass spectrometry (HPLC–MS) experiments. Starting with high lipid biomass, the bulk purification of CD1d–TCR ligators allows extensive scale-up of experiments, generating extremely high absolute sensitivity needed for whole cell lipidomics scans of CD1d–TCR bound lipid.

Analysis of CD1d eluents in each column fraction with shotgun nano-electrospray ionization-MS confirmed strong lipid-derived signals, which were similar in intensity among fractions, as expected based on protein normalization (*SI Appendix*, Figs. S2 and S3). Also, differing *m/z* values in each fraction indicated that differing ratios of CD1d–TCR versus CD1d alone generated distinct lipid capture patterns (*SI Appendix*, Fig. S3). For higher sensitivity and mass accuracy, we next implemented HPLC–time of flight–MS (HPLC–TOF–MS) with software-based (XCMS) peak picking to enumerate lipids. After censoring isotopes, salt clusters, alternate adducts, and dimers, CD1d or CD1d–TCR complexes generated 98 unique ion chromatograms with defined *m/z*, retention time, and intensity values. Comparing the early-eluting CD1d–TCR complexed fractions (31 to 33) to the CD1d alone fractions (37 to 39), these “molecular features” were ranked based on intensity ratios found in CD1d–TCR versus CD1d alone. This ranking of all CD1d captured lipids identified 15 lipids with 10-fold higher representation in CD1d–TCR complexes, which represented candidate antigens (Fig. 1*C*).

### The TCR Trap Technique Detects Known Phospholipid Antigens.

Ions differing by mass intervals corresponding to H2, CH2, and CH4 suggested the presence of saturation and chain length variants within the same ion series so that the 15 features likely corresponded to six unique lipid families (Fig. 1 *D* and *E*, colors). These six ion series matched literature values for four PIs (C34:2, C34:1, C36:2, and C36:1), two phosphatidylserines (PS, C36:2 and C36:1), and one PE (C36:1). For confirmation, one ion in each series underwent collision-induced dissociation (CID)-MS, which detected fragments matching the expected headgroups and alkyl chains (Fig. 1*D*). Notably, this screen returned lipids that matched known self-PE, PI, and PS antigens for NKT cells that were previously identified with conventional methods (14), validating that the “TCR trap” method can identify natural antigens. Eight other lipid species did not match the mass of known lipid antigens, and their structures were not solvable in negative mode mass spectrometry, which detects anions (Fig. 1*E*).

### Identifying Previously Unknown Lipids in the Positive Mode.

**Fig. 2.**
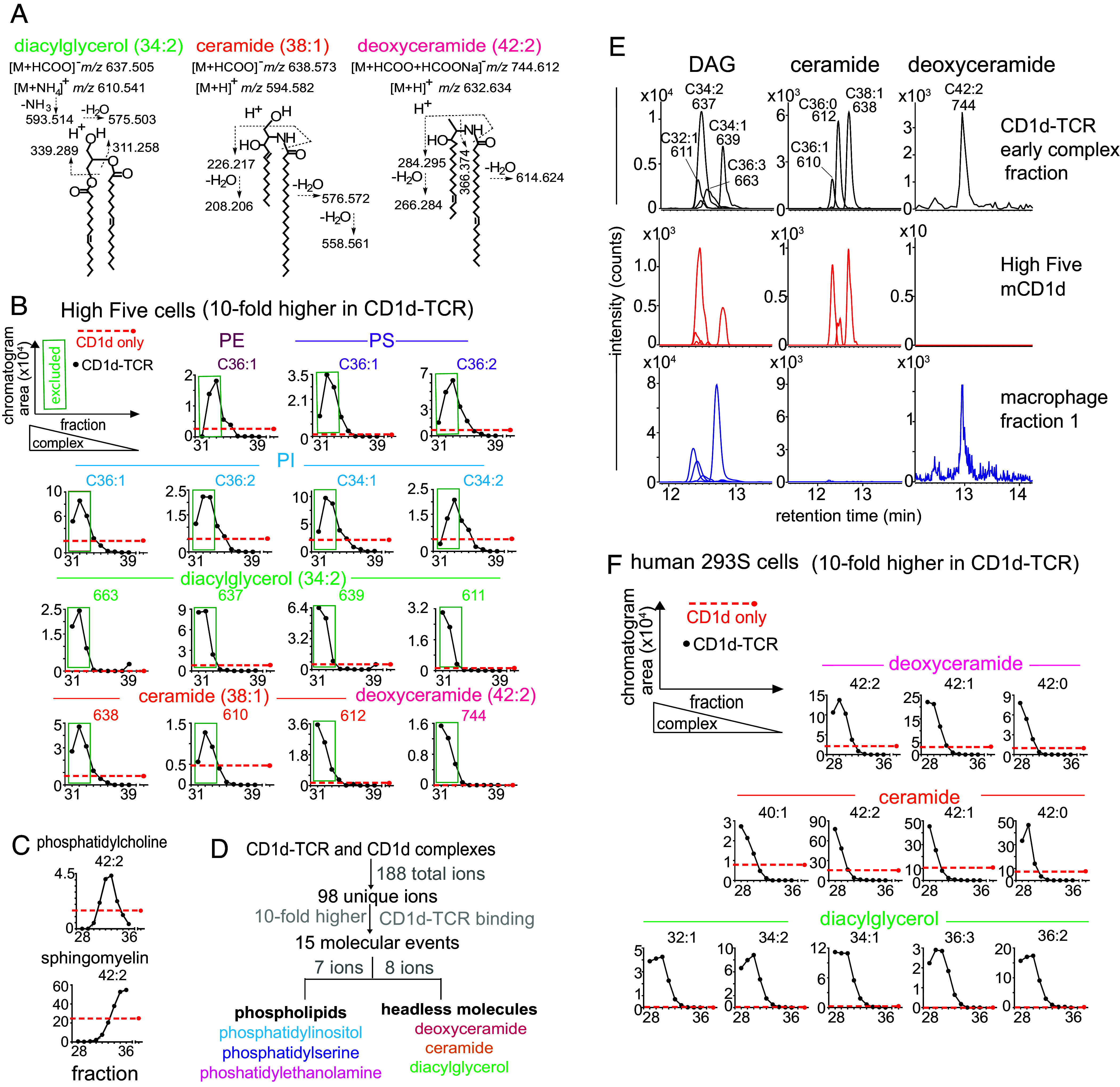
Identification of antigen structures trapped by CD1d–TCR. (*A*) Three unknowns were solved as DAG, ceramide, and deoxyceramide by matching their exact masses to literature values and by CID-MS in the positive mode. Stereochemistry and unsaturations are inferred from the literature, except for deoxyceramide, where the natural compound coeluted with a standard composed of a C1-deoxy modification and the 14*Z* unsaturation (*SI Appendix*, Fig. S4). (*B*) Fifteen named and unnamed events initially identified as being overcaptured in early fractions (31 to 33, green box) were revalidated by tracking ions across all nine fractions. (*C*) Signals matching the mass of a weak antigen (PC 42:2) and a TCR blocking lipid (SM 42:2) were tracked across fractions, demonstrating that not all lipids were included in early fractions with CD1d–TCR complexes. (*D*) A summary of eluted molecules is shown. (*E*) To determine the origin of signals from High Five (insect) and macrophage (mouse) cells, MS signals derived from CD1–TCR complexes were compared to signals from total lipid extracts from each cell source. (*F*) The TCR trap experiment was repeated by using CD1d produced in human cell line (293S) and reported as in *A*.

These eight unknown compounds in CD1d–TCR fractions clustered into three recognizable lipid series with saturation and chain length variants (Fig. 1*E*), but these unknowns simply lost the formate chemical group in collisional experiments, preventing their identification as anions. We could assign the neutral mass (M), from which separate positive mode CID-MS experiments detected cations and solved the unknowns as diacylglycerol (DAG), ceramide, and a likely deoxyceramide (Fig. 2*A*). The unusual deoxyceramide eluted from CD1d–TCR complexes was further confirmed by matching its retention time and the collisional pattern to the synthetic standard, 1-deoxyceramide [m18:1(14*Z*)/24:1(15*Z*)] but not another deoxyceramide with differing unsaturation positions (*SI Appendix*, Fig. S4). The three types of molecules differed in the presence of internal esters versus amide bonds, but all shared the lack of a phosphate or sugar headgroup. Thus, in the context of CD1 binding, where sugar or phosphate head groups normally protrude for TCR contact (24), they could be considered headless molecules. Overall, this unbiased method detected 188 total MS signals, from which 98 nonredundant signals matched 15 molecules with high CD1d–TCR binding. Considering alkane and unsaturation variants as part of the same lipid class, six lipid classes were identified, which reduced to two molecular patterns: membrane phospholipids and headless neutral lipids (Fig. 2*D*).

### Detailed Lipid Fractionation into CD1d–TCR or CD1d Fractions.

For additional validation, HPLC–TOF–MS signals were analyzed in each of the original nine fractions that contained differing ratios of CD1d–TCR to CD1d alone (Fig. 2*B*). Weak lipid signals appeared in fraction 31, which likely reflected lower overall protein capture seen in protein electrophoresis (*SI Appendix*, Fig. S2). Otherwise, all known phospholipid antigens and candidate headless antigens showed “reversed S-shaped” elution patterns with strong signals in early fractions and absent signals in late fractions. The favored interpretation was that lipids appeared in early fractions based on their ability to promote CD1d–lipid–TCR complexation, but other possibilities include direct interactions of lipids with the exclusion column matrix or nonspecific factors. Therefore, we analyzed signals corresponding to molecules that were not expected to strongly promote CD1d–TCR interactions, including phosphatidylcholine with a combined chain length of C42 with two unsaturations (42:2) and sphingomyelin 42:2. The former is a weak CD1d agonist (25) and the latter is a known blocker of CD1d–TCR interaction (26). Patterns changed such that these two lipids appeared in intermediate and late fractions, respectively, with the known TCR blocker strongly excluded from CD1d–TCR fractions (Fig. 2*C*). Thus, the profiles match the predictions that individual lipids are governed by their influence on CD1d–TCR interaction.

### Origins of Trapped Lipids in Insect and Mammalian Cells.

Because the experimental approach used lipids from two cellular sources, CD1d–TCR complexes might include lipids from either insect cells or stressed mammalian macrophages. Therefore, we sought to determine the cellular origin of TCR-trapped lipids by taking advantage of species-specific differences in the alkyl chain patterns and retention time in HPLC–MS. Overall, lipids from both cell types entered the TCR trap lipidome and were capturable. For example, TCR-trapped DAG signals matched DAG signals from both cellular sources with regard to retention time and *m/z* (Fig. 2*F*). TCR-trapped ceramides gave signals that matched those seen in High Five cells only, and deoxyceramide signals matched those from macrophage lipids only.

To focus on self-lipids from mammalian cells, we repeated the entire TCR trap experiment using CD1d produced in human embryonic kidney HEK293S (293S) cells. Here, CD1d–TCR fractions released complexes with 12 forms of DAG, ceramide, and deoxyceramides, which were not enriched in late eluting nonexcluded CD1d monomers (Fig. 2*E*). Whereas results were highly similar to those from insect cells, we did note a somewhat longer chain length (C40-42) that is characteristic of human cells (27). In summary, TCR trapping identified three previously known antigens and three kinds of headless lipids that were not expected to act as lipid antigens.

### CD1d–Deoxyceramide Binds and Activates the 2C12 TCR.

**Fig. 3.**
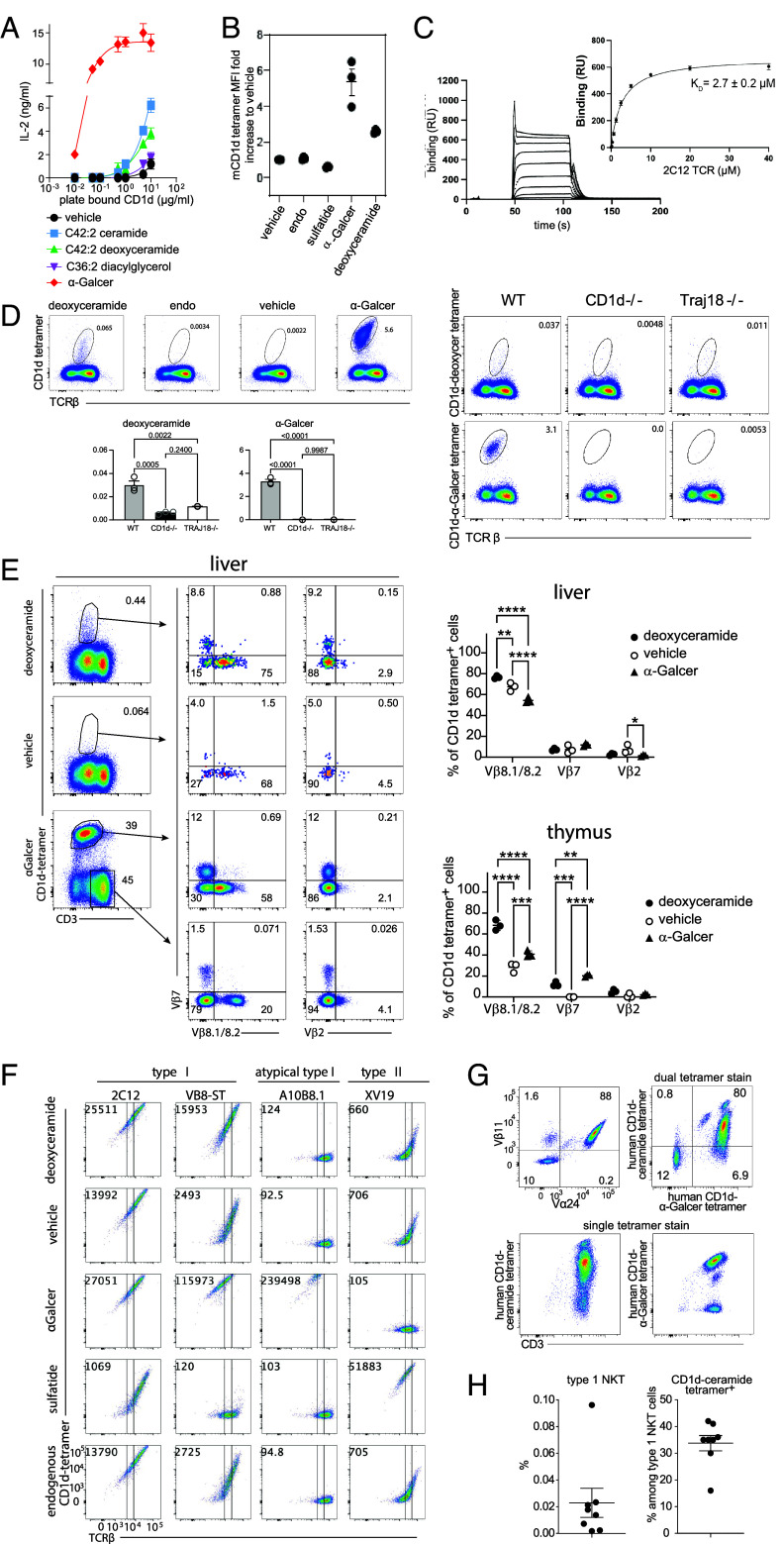
Recognition of headless antigens by NKT cells. (*A*) Activation of the 2C12 hybridoma cultured with biotinylated CD1d loaded overnight with the indicated lipid is shown for one representative experiment from two performed. Variance from vehicle alone was reported as a F-score (DAG, 0.006; deoxyceramide, 0.001; ceramide, 0.001; αGalCer 0.001). (*B*) CD1d tetramers were mock treated (endo) or treated with the indicated compound prior to staining 2C12 cells. (*C*) The equilibrium constant (K_D_) was calculated using the one-site binding model. The curve (*Left*) is representative of two independent experiments performed with technical replicates. (*D*) Thymocytes from WT mice were pooled and stained with CD1d tetramers in triplicate, where the *Left* panels show live, singlet, CD19^–^CD11b^–^CD24(HSA)^low^ cells using concatenated data from two replicates, where one of two experiments is shown. The *Right* panels show representative staining in triplicate with three thymi pooled per replicate, analyzed with a one-way ANOVA. (*E*) Representative flow cytometry plots show tetramer^+^ T cells from WT mice stained with TCR Vβ specific antibodies on cells gated to be CD19^–^CD11b^–^CD11c^–^CD8^–^CD1d endo tetramer^–^ (*SI Appendix*, Fig. S5*B*). Experiments were done in biological replicate with pooled tissues from 3 to 4 mice per replicate and analyzed with two-way ANOVA and Tukey’s multiple comparisons test. (*F*) Mouse CD1-tetramers loaded with the indicated lipid were assessed for staining of BW58 lines expressing the indicated TCRs. Representative plots from three experiments from mammalian 293S GnTI^−/−^ cells and two experiments using CD1d from High Five insect cells are shown (*SI Appendix*, Fig. S6*B*). (*G*) Previously enriched and expanded human NKT cells (*SI Appendix*, Fig. S7) were stained with antibodies against Vβ11 and Vα24, or the indicated CD1d tetramers in combination with anti-CD3. One representative experiment is shown of three independent experiments. (*H*) The frequency of type 1 NKT cells was determined by gating for CD3 and CD1d–PBS-57 tetramer^+^ cells (n = 4) or by gating for Vα24, Vβ11, and CD1d–PBS-57 tetramer^+^ cells (n = 4, *Left*) as shown in *SI Appendix*, Fig. S8 *A* and *B*. The percentage of CD1d–C42:2 deoxyceramide tetramer binding as a fraction of type 1 NKT cells is shown, where error bars represent SEM.

Next, we tested whether the headless lipids were functional antigens for NKT cells. We measured 2C12 hybridoma cell activation by treating mouse plate-bound CD1d proteins with C42:2 ceramide, C42:2 deoxyceramide, and C36:2 DAG, provided as synthetic molecules to avoid false positive responses from biological contaminants. This assay format rules out lipid interactions with APCs and instead indicates that CD1d-mediated lipid display is the mechanism of action. Compared to vehicle, both forms of ceramide were activating, and a somewhat lower but significant effect was seen for DAG (Fig. 3*A*). Similar to previously identified natural self-antigens (14, 28), the potency of these headless antigens was lower than for αGalCer.

We also investigated whether mouse CD1d tetramers treated with deoxyceramide or αGalCer stained the 2C12 hybridoma. As negative controls, we used vehicle, untreated CD1d with endogenous lipids (CD1d-endo), and sulfatide, which stains some type II NKT cells with variable TCRs (29). In agreement with the activation data, CD1d-C42:2 deoxyceramide tetramers stained 2C12 above levels seen with CD1d-endo, vehicle, and sulfatide, but staining was lower than with positive control αGalCer-treated tetramers (Fig. 3*B*). Surface plasmon resonance assays provided quantitative biophysical measurements of 2C12 TCR binding toward the CD1d–ceramide complex, finding that affinity was in the micromolar range (K_D_ ~ 2.7 µM) with fast on-and-off rates (Fig. 3*C*). These values are in the physiological range for activating antigens but are lower than the nanomolar affinity exhibited toward CD1d-bound to α–GalCer (KRN7000) (30, 31). Thus, the observed rapid kinetics corroborates the concept that NKT TCRs recognize CD1d carrying headless self-lipids, but show lower activation compared to synthetic agonists (32).

### CD1d–Deoxyceramide Stains Mouse NKT Cells.

CD1d-C42:2 deoxyceramide tetramers were used to stain thymocytes from either wildtype (WT) mice, CD1d^−/−^ mice lacking all NKT cells, and (Traj18^−/−^) mice selectively lacking type I NKT cells (Fig. 3*D* and *SI Appendix*, Fig. S5). CD1d–deoxyceramide tetramer^+^ cells were detected in WT mice, but not in the CD1d^−/−^ and were reduced in Traj18^−/−^ mice, suggesting that many of these cells were CD1d and TRAJ18 dependent, as expected for type I NKT cells. Compared to αGalCer, CD1d-C42:2 deoxyceramide tetramers stained a smaller fraction of T cells and with lower intensity.

Considering TCR patterns, liver and thymus T cells from WT mice stained with tetramers loaded with either lipid also costained with antibodies that detect TCRβ chain variable regions 8.1, 8.2, and 7 (Fig. 3*E*), again suggesting that the deoxyceramide reactive T cell population expresses TCRβ chains typical of type 1 NKT cells. However, compared to αGalCer-loaded tetramers, C42:2 deoxyceramide tetramer^+^ T cells from the liver and thymus showed bias toward Vβ8 expression, which is known to mediate higher affinity interactions with CD1d comparted to Vβ7 (31). Further testing focused on the function of individual TCRs, including those that match, partially match or do not match the type I NKT TCR motif (*SI Appendix*, Fig. S6*A*).

To further define TCRs, BW58 mouse thymoma cells were transfected with type I NKT TCRs (2C12, VB8-STD), an atypical Vα10Jα50-encoded but αGalCer-reactive TCR (A10B8.1) (33) or a type II NKT TCR that recognizes sulfatide (XV19) (29). Staining of the type I NKT TCRs with CD1d carrying mixed “endogenous lipids” (CD1d-endo) or CD1d-vehicle, suggested baseline direct reactivity for VB8-STD, and to a lesser extent 2C12, but both cell types stained more brightly when CD1d was treated with deoxyceramide (Fig. 3*F*). This effect was specific, as no augmented staining for deoxyceramide-loaded tetramers was seen for non-type I TCRs A10B8.1 and XV19, compared with CD1d-vehicle and CD1d-endo tetramers. Analysis of positive controls showed that these cell lines did, as expected, show enhanced staining by CD1d-αGalCer and CD1d sulfatide tetramers, respectively. Consistent with the lipid elution assays (Fig. 2), similar results were achieved regardless of whether CD1d was produced by insect or mammalian 293S cells (Fig. 3*F* and *SI Appendix*, Fig. S6).

Last, we tested eight BW58 lines transduced to express mouse invariant (Vα14Jα18) TCR-α chains paired with different TCR-β chains, that were previously shown to exhibit differential glycolipid reactivity (34) (*SI Appendix*, Fig. S6). Deoxyceramide consistently augmented staining (>twofold) for some (Vβ8.2-Jβ2.7n2, Vβ8.2-Jβ2.7r1, Vβ8.2-Jβ2.5r, and Vβ7-Jβ1.2r), but not all of these lines, again suggesting that deoxyceramide is detected by a subset of mouse type I NKT TCRs that differ in TCRβ.

### Human NKT Cells Recognize Deoxyceramide Compounds.

Human NKT cells are less frequent than those in mice (35), so we first sorted NKT cells from blood using human CD1d tetramer loaded with PBS-57 (36), a high potency α-GalCer analog. The resulting T cells were expanded and analyzed with anti-Vβ11 and anti-Vα24, whichbind type I NKT cell TCRs (*SI Appendix*, Fig. S7, 3g), yielding 88% Vα24^+^ Vβ11^+^ NKT cells, 10% non-NKT cells, and 1.6% Vβ11^+^Vα24^–^ T cells, which might be atypical NKT cells that lack the defining TCR but recognize αGalCer (37). CD1d–ceramide and CD1d–αGalCer tetramers stained most cells and clear cross-reactivity was seen in dual tetramer staining. Double tetramer staining was only seen when CD1d–ceramide tetramers were added first and in excess, suggesting that the interaction of human TCRs with the ceramide is weaker than αGalCer.

To study T cells directly ex vivo without first enriching for NKT cells, we stained fresh peripheral blood mononuclear cells with CD1d tetramers loaded with lipids. Low staining of non-TCR ligands with CD1d tetramers can occur (38), so we used two gating strategies that presort for type I NKT cells using anti-TCR antibodies or CD1d tetramers on PBMC from four donors for each approach (*SI Appendix*, Fig. S8 *A* and *B*). Both strategies yielded similar outcomes, with pooled data from eight donors showing that CD1d–deoxyceramide tetramers stain 33.8% of NKT cells (Fig. 3*H*). The data estimate repertoire size in human blood and again show that some ceramide-reactive T cells are a subset of type I NKT cells. Overall, human and mouse type I NKT TCRs bind to CD1d in complex with headless lipid antigens.

### Structure of CD1d Presenting Ceramide to the 2C12 TCR.

**Fig. 4.**
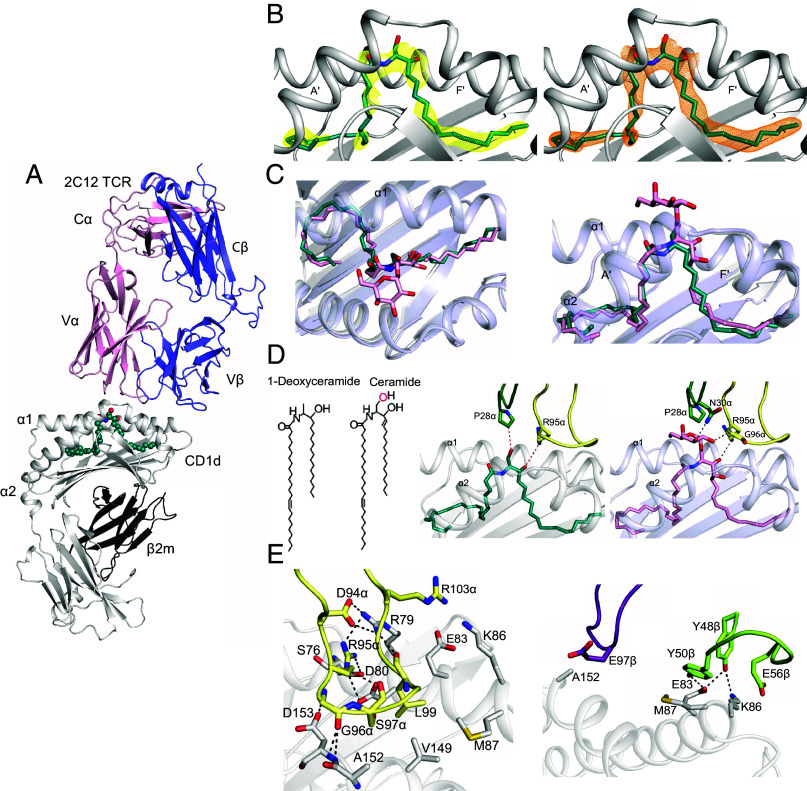
The 2C12 TCR recognizes the mouse CD1d–ceramide complex mainly by contacting CD1d. (*A*) The 2C12 TCR α- and β-chains contact CD1d–β2 microglobulin carrying ceramide represented in teal spheres. (*B*) The Fo-Fc (yellow) and 2Fo-Fc (orange) electron density maps of ceramide were contoured at 2.2 σ and 0.8 σ levels, respectively. (*C*) 2C12 TCR–CD1d–ceramide (teal) was superimposed on the 2C12 TCR–CD1d–αGalCer (KRN7000) complex (pink; PDB code: 6BNK), demonstrating the lack of a protruding head group in ceramide. (*D*) TCR α-chain interactions with ceramide (teal) or αGalCer (pink) with CDR1α and CDR3α loops colored dark green and pale yellow, respectively. Chemical structures illustrate that one of the hydroxyl groups (pink) involved in binding is absent from 1-deoxyceramide and both hydroxyl groups are missing from DAG. (*E*) TCR α-chain (*Left*) and TCR β-chain (*Right*) show that the CDR3α loop (pale yellow), CDR2β loop (green), and CDR3β loop (purple) interact extensively with CD1d with hydrogen bonds shown in dashed lines.

Most antigens have headgroups that protrude from CD1 to form TCR epitopes. To understand the molecular recognition of a neutral lipid without an obvious headgroup, we solved the ternary complex of 2C12 TCR–CD1d–C42:2 ceramide to 2.7 Å resolution (Fig. 4*A* and *SI Appendix*, Table S1). The ceramide electron density was unambiguous and complete, showing that its acyl and sphingosine chains reside in the A’ pocket and F’ pockets, respectively (Fig. 4*B*). The TCR docked in a parallel manner over the F’ portal of CD1d, similar to footprints of other NKT TCRs (24, 39). However, the current findings were notable because binding was almost exclusively mediated by contact points between the TCR and CD1d, rather than the lipid ligand.

Specifically, the antigen (Fig. 4*C*) formed a flush surface at the entry to the F’ portal. Two TCR residues, Pro28α of CDR1α and Arg95α of CDR3α formed van der Waals (vdW) interactions with the 1-OH and 3-OH of ceramide, respectively. The former contact point is lacking in deoxyceramide, and DAG has internal ester bonds instead of hydroxyl groups. Thus, these two interactions are not likely equivalent in recognition of the other headless antigens (Fig. 4*D*). Also, a hydrogen bond between the 3-OH of the sphingosine chain of ceramide interacted with Asp80 of CD1d, anchoring the lipid tail (40, 41). The superposition of ceramide and αGalCer ternary structure structures (41) showed highly similar alignments of both the acyl and sphingosine chains in both pockets (Fig. 4*C*). The αGalCer complex shows an ~800 Å^2^ footprint, versus ~650 Å^2^ for the ceramide ligand, potentially explaining the lower affinity of the ceramide antigen for the TCR.

Many individual contact points (*SI Appendix*, Table S2) formed a plane of contact of the 2C12 TCR to the outer surface of CD1d (Fig. 4*E*). The CDR3α loop mediated several hydrogen bonds located near the F’ portal, which for headgroup-containing ligands represents the antigen exit portal. These interactions involve Asp94α and Arg79, Arg95α and Asp80, Arg 95α and Ser76, Gly96α and Ala152, Gly96α and Asp153, and leu99α and Arg79 residues (Fig. 4*E* and *SI Appendix*, Table S2). The β-chain contacts to CD1d were mostly via the CDR2β and CDR3β loops with two aromatic residues of the CDR2β loop, Tyr48β and Try50β, providing hydrogen bonds with Glu83 of CD1d. Additionally, the Glu56β in the CDR2 β loop and Glu97β in the CDR3β loop mediated vdW interactions with Lys86 and Ala152 residues of CD1d, respectively (Fig. 4 *D* and *E* and *SI Appendix*, Table S2). Accordingly, nearly all of the contacts within the TCR–CD1d–ceramide complex arose from the TCR contacting CD1d, with only two vdW contacts via a nonconserved hydroxyl group lacking on other headless ligands. Many CD1d contacts occurred at the F’ portal site, which is covered by protruding headgroups in other CD1d–lipid structures, ruling in that this headless antigen provides absence of interference of TCR–CD1d contact (24).

## Discussion

Most known NKT cell antigens are bacterial in origin, yet in tissues NKT cells undergo regulation outside the context of infection in the settings of cancer, metabolic disease, and sterile inflammation (5, 6, 42). In seeking to study the spectrum of self-antigens, conventional chromatographic approaches cannot identify antigens on a cell-wide basis, because each chemically distinct molecule takes a different route through chromatographic matrices. In contrast, the TCR trap uses CD1d and TCR binding as the purification principle, allowing a single-step bulk purification of many CD1 and TCR binding compounds present in a cell. Unlike discovery methods that emphasize exogenously added lipids, this approach surveyed endogenous lipids in a system where the abundance of each lipid and the efficiency of local cellular loading interactions likely influenced outcomes (23). Whereas prior reports used TCRs to trap and analyze individual ligands (12, 21), scale-up of cellular reagents and improvements to MS sensitivity yielded a cell-wide lipidomic analysis of self-lipids.

We report 15 CD1d–TCR ligating lipids, which initially appeared to be a large number of self-lipid antigens for one TCR. However, when alkyl chain length and saturation differences are put aside, 15 molecules span six lipid families, which constitute only two general classes: amphipathic phospholipids and neutral headless lipids. One notable negative finding was that we failed to detect any glycolipid antigens, including α-hexosyl ceramide antigens, which have been proposed to occur in mammalian cells (13). However, two limitations of this otherwise broad survey are that these CD1d proteins sample the secretory, but not the endosomal recycling pathway, and tissue- or organ-specific lipids could be different than those in mouse and human cells used here. Overall, no potent or high-affinity antigens were identified in the three cell types studied. Instead, the recovery of PS, PI, and PE antigens in CD1d–lipid–TCR complexes pointed to roles of more abundant but less potent agonists (14, 32, 43). Importantly, ceramide, deoxyceramide, and DAG are three independently identified targets with one clear shared feature: lack of protruding headgroup that normally controls TCR binding. These three related antigens behave similarly in elution assays, tetramer staining, and activation assays, suggesting equivalent function on T cells.

For MHC-peptide (44, 45) and CD1-glycolipid (3, 46), early studies established the corecognition model, whereby TCRs contact both the antigen-presenting molecule and the protruding headgroups of antigens (24, 47). Small modifications of headgroup structure abrogated recognition (3, 46, 48), emphasizing the role of the TCR in precisely discerning headgroup chemistry as a means of antigen “recognition”. In contrast, here, we report that a panel defined mouse NKT TCRs, as well as polyclonal mouse and human NKT cell populations, that bind molecules that lack any headgroup. Multiple lipid antigen cross-reactivities of headless antigen-reactive TCRs with αGalCer suggested that NKT TCRs generate a framework that can bind strongly to CD1d–αGalCer or weakly to CD1d carrying self-antigens. CD1d autorecognition by NKT TCRs has been previously recognized (42, 49) and can occur when the TCR binds the CD1d surface in ahydrophobic patch that isjust adjacent the protruding headgroup (32). Here, the mechanism involves lack of protrusion of any headgroup. Undermining the general nature of the corecognition paradigm, the agonist acts not through precise TCR discernment of its headgroup, but instead by not interfering with intrinsic CD1d–TCR autoreactivity.

The CD1d–lipid–TCR structures provide a clear and detailed view of this unexpected mode of NKT TCR binding. Unlike αGalCer, ceramide sits flush with the antigen portal, allowing the 2C12 TCR to closely approach and extensively engage CD1d, where ~20 TCR–CD1d interactions form along the CD1d–TCR interface. These are located near to what is normally the antigen exit portal, but in this case, the antigen does not significantly protrude. The TCR–galactose interactions seen for αGalCer are absent for ceramide, contributing to the smaller TCR footprint and likely to the lower affinity. Although two bonds were seen between the TCR and ceramide, they are unlikely to be essential for headless antigen recognition in general, since deoxyceramide lacks the 1-OH group and both interacting hydroxyls are absent in DAG. These structures show how abundant but highly regulated self-ceramides act by absence of interference, where the headless nature of ceramides allows a close type I NKT TCR approach to CD1d. We did not detect augmentation of CD1d binding to the type II NKT TCR XV19 by ceramides. However, type II NKT with diverse TCRs are not ruled out and should be further explored since TRAJ18 deletion did not fully delete ceramide-reactive cells and headless antigens have been also detected for diverse TCRs recognizing CD1a (21, 50) and CD1c (51).

Early work on potent synthetic or bacterial hexosyl ceramides suggested that ceramide is not an agonist for NKT cells (3), which contrasts with our data. This outcome may reflect the low doses tested, the low potency of ceramide antigens, or differences in ceramide chain length in the studies. Indeed, our data support that the headless ligands are weakly acting agonists compared to αGalCer. However, the measured affinity of 2C12 for CD1d–ceramide is in the physiological affinity range for activating TCRs. Also, whereas our studies of NKT cell hybridomas exposed to plate-bound proteins or transfected thymoma cells showed low potency response to ceramides, ceramides are quite abundant in cells. Further, in previously published studies of macrophage activation by ER stress signals (5, 6), as well as studies of total macrophage lipids from thapsigargin-treated cells used here, strong T cell response was seen. In some cases, response was mediated by cytoskeletal changes occurring in stressed macrophages (5), suggesting that headless antigen generation and CD1-independent aspects of APC activation can be simultaneously induced and may represent synergistic signals. Overall, these data contribute to an updated view of NKT cell response that emphasizes two general types of antigens: self-antigens with high cellular abundance and low potency or foreign antigens with low abundance or restricted expression and high potency. Prior work has emphasized potent bacterial α-hexosyl glycolipids (7, 11) or intestinal symbionts (9), and milk antigens (12), but their entry into uninfected normal tissues is undocumented. Here, abundant self-phospholipids and headless lipids constitute the fifteen top targets for CD1d–TCR ligation, and such self-phospholipids, lysophospholipids (14), and headless hydrophobic antigens are broadly present in mammalian cells. Weak agonism can have important impacts in positive selection in both the MHC and CD1 systems. Ceramides are abundant, highly regulated, and efficiently captured by CD1d based on several lines of evidence, including high ceramide accumulation in CD1d-expressing gastrointestinal tissues during infection (52), high capture of endogenous ceramides by CD1d in cells (23) and our TCR trap data.

Looking forward, headless lipids, acting as ubiquitous and highly regulated antigens, which are weaker when compared to αGalCer, emerge as candidates for regulating positive selection of NKT cells. Further, our data provide molecular links to induction of headless antigens in defined cell stress pathways. While the genesis of headless antigens in CD1d–TCR complexes was not directly established here, prior evidence for ceramide induction by thapsigargin (53), detection of deoxyceramides in Fraction 1, and the high signals in thapsigargin-treated macrophages were consistent with PERK and IRE-1a playing a role in antigen induction. Ceramide and sphingomyelin are the two central intermediates in the sphingomyelin cycle (16) that are oppositely regulated in apoptosis and transformation (54, 55). Also, ceramide strongly accumulates in gastrointestinal cells after caspase activation during bacterial infection (52). Recent data show (23, 26, 56) that CD1d captures sphingomyelin to block NKT cell activation (26, 56). Thus, one major upstream stress signal, acting through the sphingomyelin cycle can simultaneously deplete sphingomyelin blockers and generate ceramide antigens for NKT cells. Future work will move beyond thapsigargin to investigate natural stressors that control the balance of NKT cell inhibitory sphingomyelins and stimulatory ceramides.

## Materials and Methods

Detailed methods are found in *SI Appendix* section.

### CD1d–TCR Complex Formation.

The His-T tagged CD1d ectodomain harboring a His-6 tag was produced in baculovirus (57) and expressed in High Five cells or 293S with or without the N-acetylglucosaminyltransferase-I gene (*GnTI−*) (58). The genes encoding the 2C12 TCR chains were expressed in *Escherichia coli* (59) and refolded. A purification process involving ditheylethanolamine anion exchange, size exclusion, anion exchange (Hitrap Q), and hydrophobic interaction chromatography was carried out, followed by sodium dodecylsulfate polyacrylamide gel electrophoresis as previously described (44). In trap experiments, Fraction 1 of thapsigargin-treated J774.2 macrophages was incubated with soluble mouse CD1d for 16 h. The 2C12 TCR–CD1d–ceramide ternary complex for crystallization was generated in a similar manner using synthetic C42:2 ceramide.

### Lipid Analysis.

After lipid extraction using the Bligh and Dyer method, CD1 protein eluents were analyzed by shotgun nano-electrospray ionization(ESI)-MS and reversed-phase HPLC ESI–QToF–MS (60). Lipids with >10-fold higher recovery in the cocomplex fractions were solved by CID-MS, using synthetic standards where available.

### Immunology Assays.

C57BL/6 mice were purchased from Jackson Laboratory (USA). Mouse cells, the 2C12 hybridoma, and BW5147.TCR α^−^β^−^ thymoma cells (BW58) transduced with TCRs were studied with anti-mouse antibodies, mouse CD1d tetramers, and 7-aminoactinomycin D fixable viability dye. For activation assays, lipids were mixed with mouse CD1d (NIH Tetramer Facility) and then added onto avidin-coated 96-well plates for 1 h prior to IL-2 enzyme linked immunosorbent assay. Human PBMC were obtained from leukoreduction collars, as approved by the Partners Healthcare Institutional Review Board (IRB), Boston. Informed consent was not required for use of discarded material under IRB oversight. Human PBMC were sorted for double positive staining of CD3 and CD1d–PBS-57 tetramer and expanded for 14 to 18 d with allogeneic PBMCs, transformed B cells, OKT3, and 2 nM IL-2. Human tetramers from the NIH Tetramer Core Facility carrying endogenous lipids (CD1d-endo) were sonicated in PBS with 0.05% Tween-20 at 37 °C followed by tetramerization with streptavidin-phycoerythrin. For primary T cells, OKT3 was added for 10 min prior to analysis.

### Crystallography.

As reported (61), crystals were grown using the hanging drop vapor diffusion method at 4 °C followed by flash freezing in 10% glycerol. Diffraction data were collected at the Australian Synchrotron with molecular replacement using the PHASER-MR, Phenix, crystallographic object-oriented toolkit, and PyMOL programs. Affinity measurements were completed using a Biacore 3000 instrument with biotinylated CD1d loaded onto a streptavidin chip, followed by passing the 2C12 TCR at a flow rate of 5 μL/min.

## Supplementary Material

Appendix 01 (PDF)

## Data Availability

All study data are included in the article and/or *SI Appendix*.
