# Supplementary material for: Lipidomic scanning of self-lipids identifies headless antigens for natural killer T cells
Source: Proc Natl Acad Sci U S A. 2024 Aug 14;121(34):e2321686121. doi: 10.1073/pnas.2321686121 (PMC11348285; doi:10.1073/pnas.2321686121)
Supplement: Supplementary file 1 — Appendix 01 (PDF) [file pnas.2321686121.sapp.pdf]

Supporting Information for:

Lipidomic scanning of self lipids identifies headless antigens for NKT cells.

Tan-Yun Cheng, T. Praveena, Srinath Govindarajan, Catarina F. Almeida, Daniel G. Pellicci, Wellington C. Arkins, Ildiko Van Rhijn, Koen, Venken, Dirk Elewaut, Dale I. Godfrey, Jamie Rossjohn, D. Branch Moody

Primary contact bmoody@bwh.harvard.edu; Hale 6002K, 60 Fenwood Road, Boston MA 02115 USA

This PDF file includes:

Supporting Text
Figures S1 to S8
Tables S1 to S2
SI References

Supporting Information Text

Extended materials and methods.

Cells. The 2C12 hybridoma, as well as the BW5147.TCR $\alpha^-\beta^-$ thymoma cells (BW58) transduced with the 2C12, VB8-STD, A10.B8.1, Vβ8.2-Jβ2.7n2, Vβ8.2-Jβ2.7r1, Vβ8.2-Jβ2.7n1, Vβ8.2-Jβ2.4n2, Vβ8.2-Jβ2.7r2, Vβ8.2-Jβ2.4n1, Vβ8.2-Jβ2.5r, Vβ7-Jβ1.2n and Vβ7-Jβ1.2r (1), or XV19 TCRs were cultured in RPMI-1640 supplemented with 10% (v/v) heat inactivated fetal bovine serum, 2% (v/v) human AB serum (Sigma), penicillin-streptomycin (100 U/ml, Sigma), Glutamax (2 mM), sodium pyruvate (#1 mM), nonessential amino acids (0.1 mM), HEPES buffer (15 mM, pH 7.2-7.5), all from Thermo Fisher Scientific, and 2-mercaptoethanol (50 µM, Sigma), at 37 °C, 5% CO_2.

Human PBMC were obtained from leukoreduction collars, as approved by the Partners Healthcare Institutional Review Board, Boston, USA, Protocol #:2002P000057. PBMCs were sorted for double positive staining of CD3 and CD1d-PBS-57 tetramer. Sorted T cells were expanded for 14-18 days with 25 × 10^6 irradiated, allogeneic PBMCs and 5 × 10^6 irradiated Epstein-Barr virus-transformed B cells per 10^6 sorted cells, in the presence of 30 ng/ml OKT3 and 2 nM IL-2 added on day 2 of the culture.

Protein production. The truncated mouse CD1d ectodomain harboring a C-terminus His-6 tag (GSGLNDIFEAQKIEWHEHHHHHH) and a C-terminus biotinylation tag, along with β2M was cloned into the pBacp10pH vector and the recombinant protein was produced in the baculovirus expression system (2). In brief, 1% of P3 viral stock transfected 1 litre of High Five cells. The secreted protein was harvested after 72 hours and dialysed against 10 mM Tris pH 8.0 and 150 mM NaCl buffer for 16 hrs. Alternatively, mouse CD1d was produced in 293S Human Embryonic Kidney cells (293S) with or without the N-acetylglucosaminyltransferase-I gene (HEK-293S.*GnTI*−) by co-transfection with pHLsec vectors encoding truncated mouse CD1d ectodomain with a C-terminal biotinylation motif and His6-tag, or β2-microglobulin, using polyethylenimine essentially as described (3). The protein was purified using a two-step purification protocol including conventional Nickel Transfer Agarose (Ni-NTA) and size exclusion chromatography (4, 5).

The genes encoding TCRα and TCRβ chains of the 2C12 TCR were codon optimised, synthesized (Genscript) and cloned into pET30 (Novagen) for expression in *Escherichia coli* strain BL21 and purified as inclusion bodies (IBs) (6). Inclusion bodies (120 mg and144 mg of α:β chain refold), in the presence of 1 mM DTT, were resuspended in 8 M urea 1000 mM Tris-HCl-pH 8.0, 0.2 mM Na-EDTA, 0.4 M arginine, 0.5 mM oxidised glutathione, 5 mM reduced glutathione and 0.2 M phenylmehtylsufonyl fluoride and X pepstatin, stirred at 4°C for 24 hrs, dialysed in 10 mM Tris-HCl buffer with three changes over 16 hrs. A four-step purification process involving DEAE anion exchange, size exclusion, anion exchange (Hitrap Q) and Hydrophobic Interaction

chromatography (HIC) was carried out, followed by SDS-PAGE gel electrophoresis under reducing and non-reducing conditions (7).

CD1d-TCR complex formation. Fraction 1 of lipids extracted from thapsigargin treated J774.2 macrophages was solubilized in 0.5% v/v tyloxapol in (TBS, pH8.0). Solubilized fraction 1 was incubated with insect cell or mammalian derived soluble mCD1d in a 6:1 lipid:protein molar ratio for 16 hours at room temperature and passed over a Superdex 200 16/60 column (GE Healthcare) using TBS, pH8.0, to remove excess detergent. The TCR trap assay (8, 9) was performed by mixing a 1:1 molar ratio of mCD1d containing lipids from fraction 1 with soluble 2C12 TCR, and then passage over a Superdex 200 16/60 column in TBS, at a flow rate of 0.5mls/min. Fractions obtained from the gel filtration column were analysed by SDS-PAGE gel.

The generation of 2C12 TCR-CD1d-ceramide ternary complex for crystallization was generated in a similar manner. In brief, C42:2 ceramide was solubilized in 0.5% v/v tyloxapol and mixed with mCD1d in a 5:1 molar ratio, prior to incubation at room temperature overnight. Excess tyloxapol detergent was removed from mCD1d using a Superdex 300 10/300 column (GE Healthcare), and mCD1d loaded containing ceramide was mixed with the 2C12 TCR in a 1:1 molar ratio and incubated overnight at 4°C. Ternary complex was isolated by performing a size exclusion chromatography (S200 16/60) (GE Healthcare).

Lipid extractions and analysis. CD1 protein fractions were extracted of lipids by the Bligh and Dyer method. For shotgun nano-ESI-MS*,* extracted lipids were analysed on a Thermo Fisher LXQ Ion Trap in the negative ion mode. Eluents were normalized to 10 µM based on protein input and 10 µL were loaded on a reversed-phase HPLC column Agilent Poroshell EC-C18 column (1.9-micron, 3 x 50 mm) with a guard column (3 x 5 mm, 2.7 µm; 1260 HPLC system) and monitored with Agilent 6530 Accurate-Mass ESI-QToF-MS using previously published gradient (10). Protein normalized eluents were loaded on a reversed-phase HPLC column and monitored with ESI-QToF-MS. Lipids from early eluting co-complexes enriched for CD1d-TCR were compared late fractions containing CD1d and TCR monomers, to identify targets whose intensity was >10-fold higher in the co-complex fractions. We removed the redundantly detected isotopes and alternate adducts, as well as salt clusters identified based on low mass defects, yielding 15 molecular events in 6 lipid classes as the TCR-trap antigens. Similar results were obtained using High Five insect cells or human 293S cells.

Lipid standards. 1-deoxyceramide (m18:1(14Z)/24:1(15Z)) 42:2 deoxyceramide, cat# 27563) used for analytical chemistry, bioassays, and tetramer staining, was purchased from Cayman Chemical. Ceramide (d18:1/24:1(15Z)) 42:2 ceramide, 860525), 1-deoxyceramide (m18:1(4E)/24:1(15Z), 860457, used for analytical chemistry) and DAG (18:1/18:1, 800811) were purchased from Avanti Polar Lipids.

Mice. C57BL/6, Traj18 - /- and CD1d1/2 -/- mice, all on C57BL/6 background, were purchased from Jackson Laboratory (USA). All mice were maintained under specific pathogen-free conditions in the Animal Care Facility of Ghent University as approved by the Institutional Animal Care and Ethics Committee**.** Experiments were performed with age- and sex-matched mice at 10–12 weeks of age. Cell suspensions from the thymus were resuspended in PBS containing 1 mM ethylenediaminetetraacetic acid and 0.5% BSA.

Flow cytometry and antibodies. Murine thymocytes were isolated as described (11). Liver cells were isolated based on Percoll density gradient centrifugation (Amersham) as described (12). For detection and enumeration of mouse CD1d tetramer binding T cells among thymocytes, cells were incubated with a viability dye (Fixable Viability Stain 700; BD), followed by mCD1d tetramer - loaded with either C42:2 deoxyceramide (Cayman #27563),, αGalCer or vehicle or left untreated (endo) – and Fc-receptor block (Miltenyi) prior to surface staining with mAb directed against CD11b (PerCpcy5.5), CD19 (PerCpcy5.5), TCRβ (BV500; all from BD), CD24 (FITC; Biolegend). For TCRVβ profiling of mouse NKT cells, thymic and liver cells were incubated with Fixable Viability Stain 700, followed by mCD1d tetramer (loaded with either deoxyceramide, αGalCer or vehicle) and Fc-receptor block (Miltenyi Biotec) prior to surface staining with mAb directed against Vβ2 TCR (BV711), Vβ8.1/8.2 TCR (BV605), Vβ7 TCR (FITC), CD19 (PERCP5.5), CD11b (PERCP5.5), CD11c (PERCP5.5; all from BD), CD24 (APC), CD8 (PE-Vio770), CD3E (APC-Vio770; all from Miltenyi) and mCD1d endo-loaded tetramer (BV421). Mouse CD1d biotinylated monomers were provided by the NIH tetramer core facility (Atlanta, USA), in house loaded (with indicated lipids) and tetramerized with streptavidin (SA)-PE or BV421 (BD) following standard protocols. Cell acquisition was performed on a 4-laser BD LSRFortessa™ and flow cytometric data were analysed using FlowJo software v.10.8.1 (BD). For TCR transduced cell staining with anti-TCRβ (APC or BV711), 7AAD live/dead marker and CD1d tetramers, mouse CD1d proteins produced in house in the Godfrey laboratory were used, loaded with PBS-44 αGalCer (a kind gift from Paul Savage), sulfatide C24:1 (Avanti Polar Lipids. #860571), each loaded at 6:1 lipid:CD1d molar ratio, or C42:2 deoxyceramide (Cayman #27563), loaded at 24:1.

For human tetramers, pre-assembled human unloaded and CD1d-PBS-57-loaded tetramers and biotinylated CD1d monomers were obtained from the NIH Tetramer Core Facility. C42:2 ceramide from Avanti (#860525) was sonicated for 1 hour in PBS with 0.05% Tween-20 at 37°C before mixing with CD1d monomers a 40:1 molar ratio lipids:CD1d. The loading was performed overnight at 37°C followed by tetramerization with SA-phycoerythrin (PE) or SA-APC (Invitrogen). Tetramers (1 μl per 50 μl cell suspension) were incubated for 20 min at room temperature before addition of antibodies. For staining of primary T cells, 0.1 μg of unlabeled monoclonal anti CD3 antibody (OKT3) was added during the last 10 min of the incubation of the

CD1d tetramers. Subsequently, fluorescently labelled antibodies were added, including CD3-fluorescein isothiocyanate (FITC) clone SK6 (Beckton Dickinson), Vβ11-FITC (Beckman Coulter), and Vα24-PerCP-Cyanine5.5 (Biolegend). Cells were washed and resuspended in PBS with 2% bovine serum albumin (BSA) and analyzed on a 5-laser LSR Fortessa flow cytometer (BD Biosciences) with FlowJo software.

CD1d-plate assay: lipids were mixed with biotinylated CD1d (NIH Tetramer Facility) in Tris buffer pH 8.0 overnight then added onto avidin coated 96 well plates for 1 h at room temperature, washed twice with PBS before adding 50,000 N38-2C12 hybridoma cells in complete DMEM medium with β-mercaptoethanol medium for 16h incubation at 37°C and 5% CO2 and collecting supernatants for IL-2 measurement by ELISA.

X-ray crystallography and structure determination. The isolated ternary complex fractions were pooled and concentrated to 5 mg/ml. Using reported methods (13), crystals were grown in conditions containing 12-16% PEG 3350, 8% Tacsimate, pH 5.0 using the hanging drop vapour diffusion method at 4°C. Crystals with good morphology were flash frozen in the mother liquor containing 10% glycerol as a cryoprotectant. The crystal diffraction data were collected at the MX2 beamline at the Australian Synchrotron. The data were processed with the program XDS (14) and scaled using SCALA (15) in the CCP4 program suite. Using 2C12 TCR and mCD1d without the lipid in the 2C12 TCR-mCD1d-KRN7000 (αGalCer) complex (PDB 6BNK) as two independent search ensembles, the structure solution was obtained by molecular replacement with the help of PHASER-MR program (16). An initial rigid body refinement followed by iterative rounds of refinement was performed with the Phenix program. Model building of amino acid residues was carried out in COOT prior to running the refinement cycles (PyMOL Molecular Graphics System, version 2.0).

Surface Plasmon resonance (SPR): Affinity measurements were carried out at 25°C using a buffer containing 10 mM Hepes-HCl, pH 8.0 and 150 mM NaCl on a Biacore 3000 instrument. The biotinylated mouse CD1d monomers loaded with ceramide were immobilised on the streptavidin (SA) sensor chip to ~2500 response units. Then, 2C12 TCR was passed over the chip at a flow rate of 5μl/min with increasing concentrations from 0-40 μM. Using a one-site binding model, the equilibrium dissociation constant (K_D) was calculated using Graph pad prism software 6.0d.

Statistics: Statistical analyses were performed using Genstat V.19 and GraphPad Prism V.9. For the plate binding assay, an exponential curve of the form y = A + B*R X was fitted to the data, where A (asymptote), A-B (starting point when X = 0), and R (speed at which the asymptote is approached), and X (concentration) generate an accumulated analysis of variance for each pair of compounds, using the F-test.

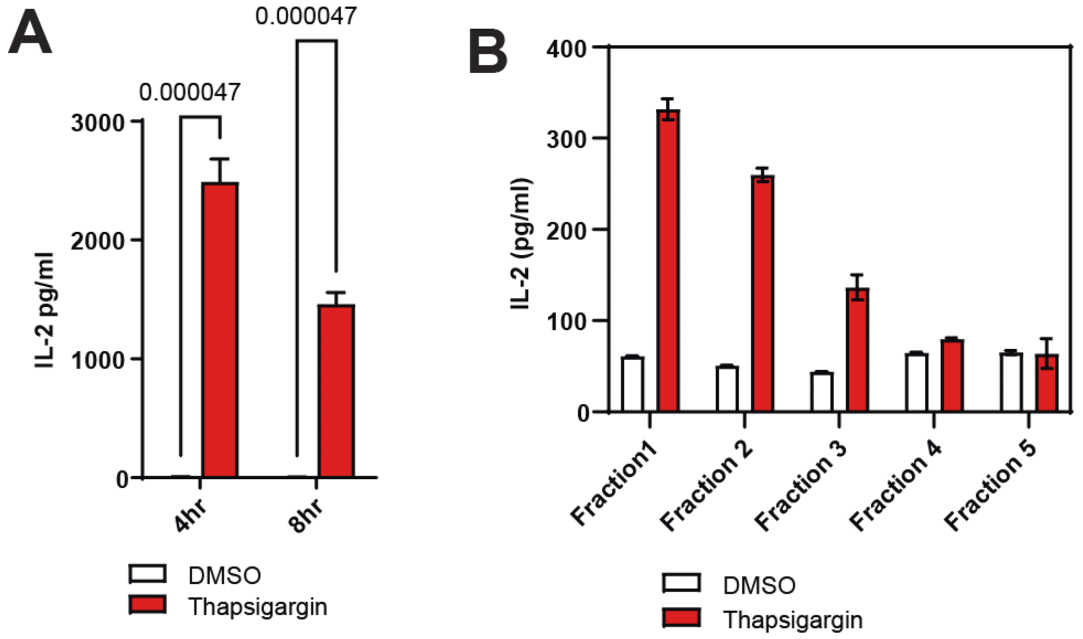

Fig. S1. Thapsigargin-treated macrophages activate NKT cells (A) Bar graph represents IL-2 secretion by NKT cell hybridoma (2C12) co-cultured with a murine macrophage cell line (J774.2) treated with either thapsigargin (red bars), or DMSO (white bars). Graphs show mean ± SEM from n = 2 biological replicates combined. Results were analysed by unpaired t-test. (B) Bar graph represents increased 2C12 activation by CD1d protein-bound neutral lipid-containing fractions (fractions 1-3) isolated from thapsigargin (red bars) compared to DMSO (white bars) treated J774.2 cells. Polar lipid-containing fractions (fractions 4-5) induce very little IL-2 secretion by 2C12. Graphs show mean ± SEM.

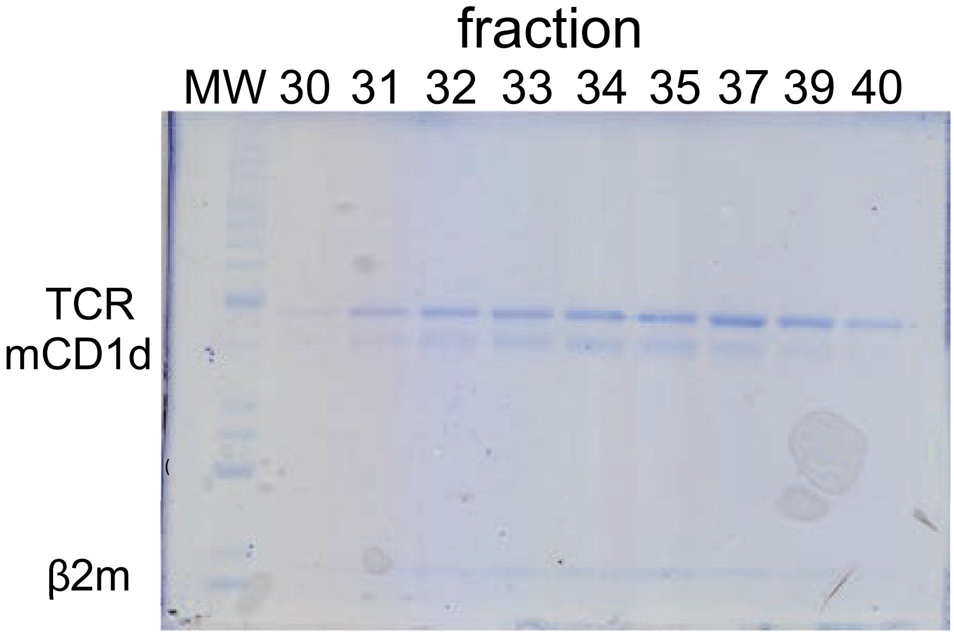

Fig. S2. Protein analysis of fractions CD1d from High Five cells and 2C12 TCR heterodimers were mixed in equimolar ratios and loaded onto a size exclusion column, collected as fractions and subjected to polyacrylimide gel electrophoresis to estimate molar ratios and protein yields needed to normalize lipid fractions.

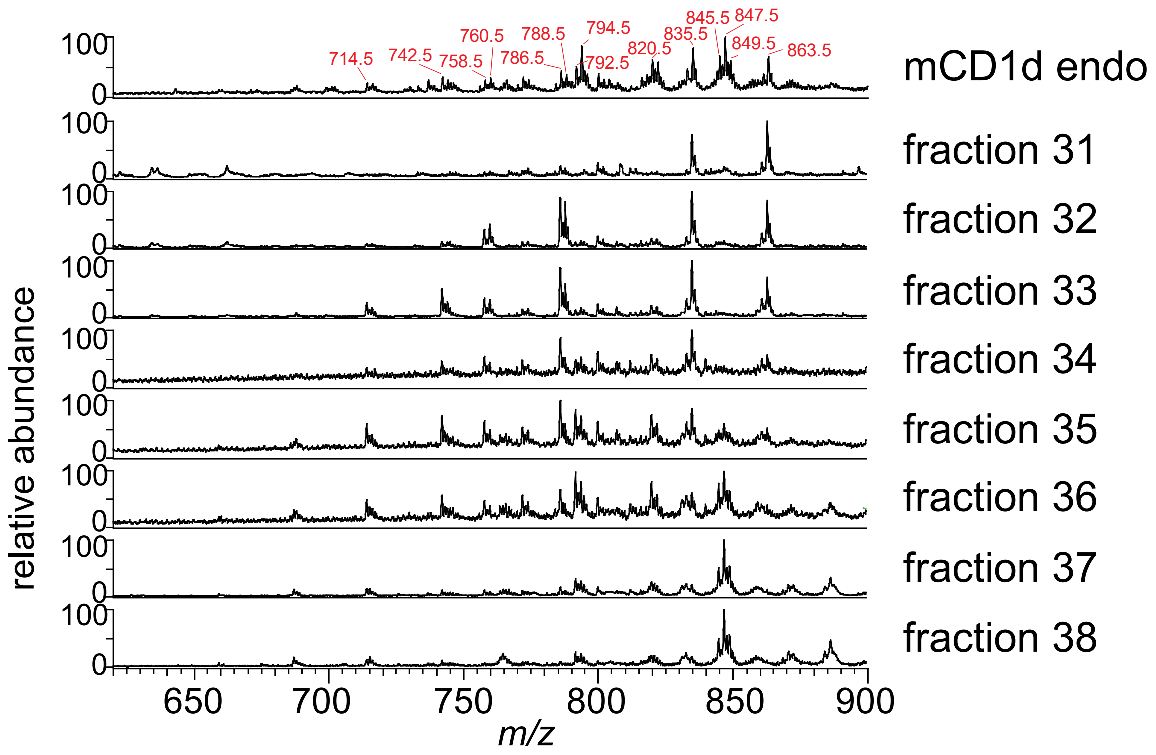

Fig. S3. Nano-electrospray ionization mass spectrometry (nano-ESI) analysis of CD1d bound lipids. Total mouse CD1d (mCD1d) expressed in insect High Five cell line was captured and treated with chloroform and methanol to detect eluted lipids by negative mode nano-ESI MS, finding numerous bright ions corresponding to endogenous CD1d lipids (mCD1d-endo) loaded in cells. mCD1d-endo was treated with macrophage neutral lipids (fraction 1 purified from thapsigargin treatment) and then mixed with recombinant 2C12 TCR and subjected to size exclusion chromatography to detect CD1d-TCR complexes in early (31-33) and CD1d and TCR monomers in late (37-38) fractions, which were simultaneously monitored by nano-ESI-MS.

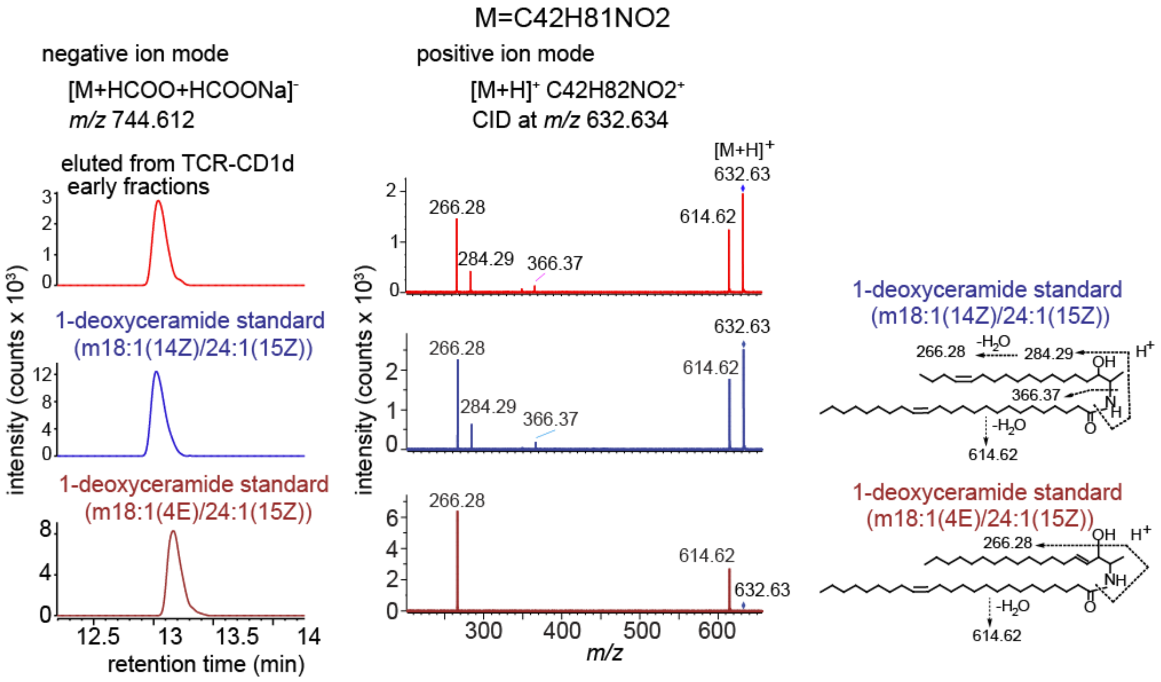

Fig. S4. Structure identification of unknown lipid antigen 744. The unknown lipid antigen 744 (top panels, m/z 744.612 in the negative ion mode; m/z 632.634 in the positive mode) was identified as deoxyceramide. The structure details were established by the exact mass, retention time, and the collisional pattern matching to the authentic molecule, 1-deoxyceramide consisting of m18:1(14Z)/24:1(15Z) (middle panels), but not m18:1(4E)/24:1(15Z) (bottom panels).

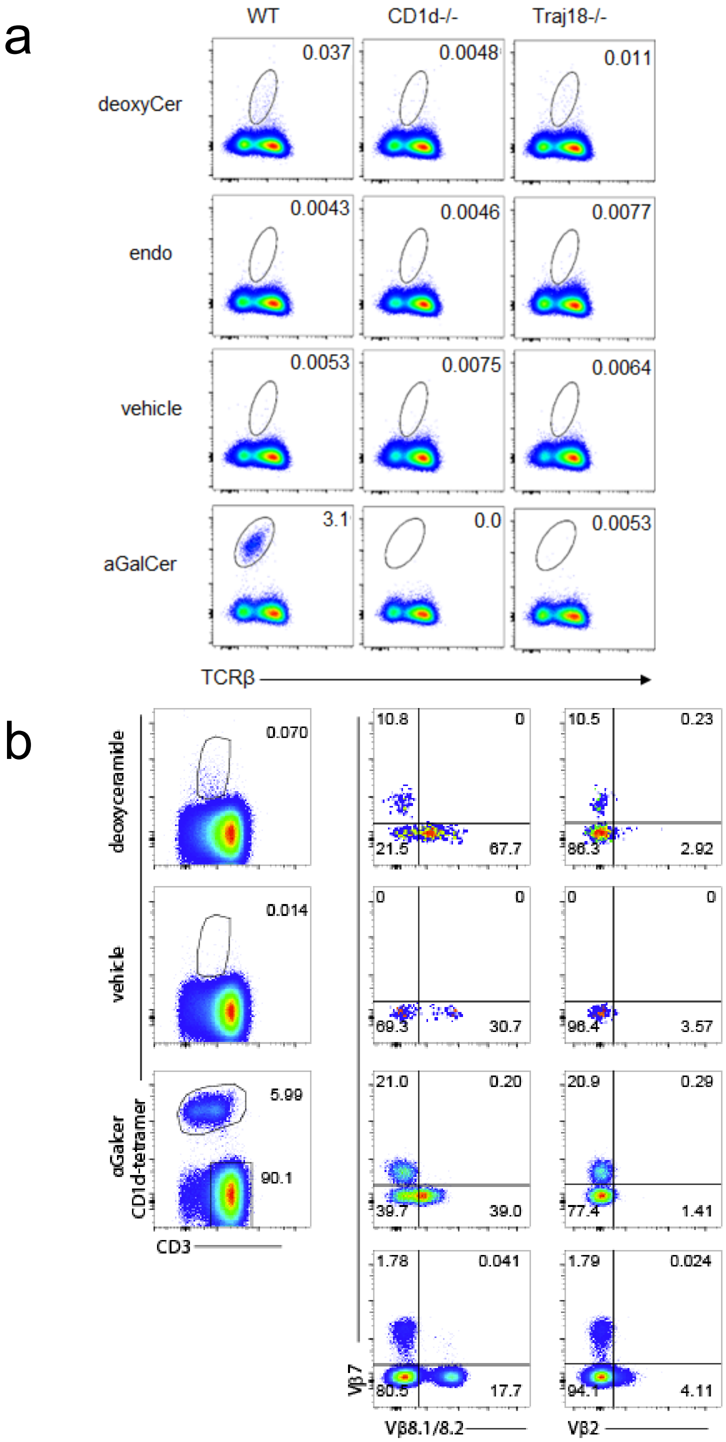

Fig. S5. Detection of murine deoxyceramide-specific cells ex vivo. Thymocytes from wild-type C57BL/6 mice were pooled and aliquoted into homogenised cellular fractions, and a) stained with PE conjugated mCD1d tetramers that were untreated (CD1d-endo), or loaded with 42:2 deoxyceramide, αGalCer or vehicle, analyzed in biological triplicate per tetramer condition. Dotplots show live, singlet, $CD19^{-}CD11b^{-}CD24^{low}$ thymocytes as concatenated data derived from two replicates per condition. b) Representative flow cytometry plots showing Vβ2, Vβ7, and Vβ8.1/8.2 staining of tetramer^{+} thymocytes after gating for $CD19^{-}CD11b^{-}CD11c^{-}CD8^{-}CD24^{low}$ phenotype. These stainings form the basis for the quantification overview in Fig. 3e.

a

NKT	Clone	Vα	V-N	Jα	Vβ	V-NDN	Jβ
Type I	**2C12**	Vα14	CVVG	DRGSALGRLHFGAGTQLIVIP (Jα18)	Vβ8.2	CASGD**EGY**	TQYFGPGTRLLVL (Jβ2.5)
Type I	**VB8-STD**	Vα14	CVVG	DRGSALGRLHFGAGTQLIVIP (Jα18)	Vβ8.2	CASGD**AGG**	NYAEQFFGPGTRLLTVL (Jβ2.1)
Atypical Type I	**A10B8.1**	Vα10	CAIAS	SSFSKLVFGQGTSLSWP (Jα50)	Vβ8.1	CASR**LGG**	YEQYFGPGTRLTVL (Jb2.7)
Type II	**XV19**	Vα1	CAAS**EQ**	NNYAQGLTFGLGTRVSVFP (Jα26)	Vβ16	CASS**FWGA**	YAEQFFGPGTRLLTVL (Jβ2.1)
Type I	**Vβ8.2-Jβ2.7n1**	Vα14	CVVG	DRGSALGRLHFGAGTQLIVIP (Jα18)	Vβ8.2	CASGDG**TGV**	YEQYFGPGTRLTVL (Jβ2.7)
Type I	**Vβ8.2-Jβ2.7n2**	Vα14	CVVG	DRGSALGRLHFGAGTQLIVIP (Jα18)	Vβ8.2	CASGD**NELGA**	YEQYFGPGTRLTVL (Jβ2.7)
Type I	**Vβ8.2-Jβ2.7r1**	Vα14	CVVG	DRGSALGRLHFGAGTQLIVIP (Jα18)	Vβ8.2	CASGD**N**	SYEQYFGPGTRLTVL (Jβ2.7)
Type I	**Vβ8.2-Jβ2.7r2**	Vα14	CVVG	DRGSALGRLHFGAGTQLIVIP (Jα18)	Vβ8.2	CASGD**ALGES**	SYEQYFGPGTRLTVL (Jβ2.7)
Type I	**Vβ8.2-Jβ2.4n1**	Vα14	CVVG	DRGSALGRLHFGAGTQLIVIP (Jα18)	Vβ8.2	CASGD**N**	SQNTLYFGAGTRLSVL (Jβ2.4)
Type I	**Vβ8.2-Jβ2.4n2**	Vα14	CVVG	DRGSALGRLHFGAGTQLIVIP (Jα18)	Vβ8.2	CASGD**WGEG**	QNTLYFGAGTRLSVL (Jβ2.4)
Type I	**Vβ8.2-Jβ2.5r**	Vα14	CVVG	DRGSALGRLHFGAGTQLIVIP (Jα18)	Vβ8.2	CASG**EG**	DTQYFGPGTRLLVL (Jβ2.5)
Type I	**Vβ7-Jβ1.2n**	Vα14	CVVG	DRGSALGRLHFGAGTQLIVIP (Jα18)	Vβ7	CASSS**PGQGP**	DYTFGSGTRLLVI (Jβ1.2)
Type I	**Vβ7-Jβ1.2r**	Vα14	CVVG	DRGSALGRLHFGAGTQLIVIP (Jα18)	Vβ7	CASS**PDRGH**	SDYTFGSGTRLLVI (Jβ1.2)

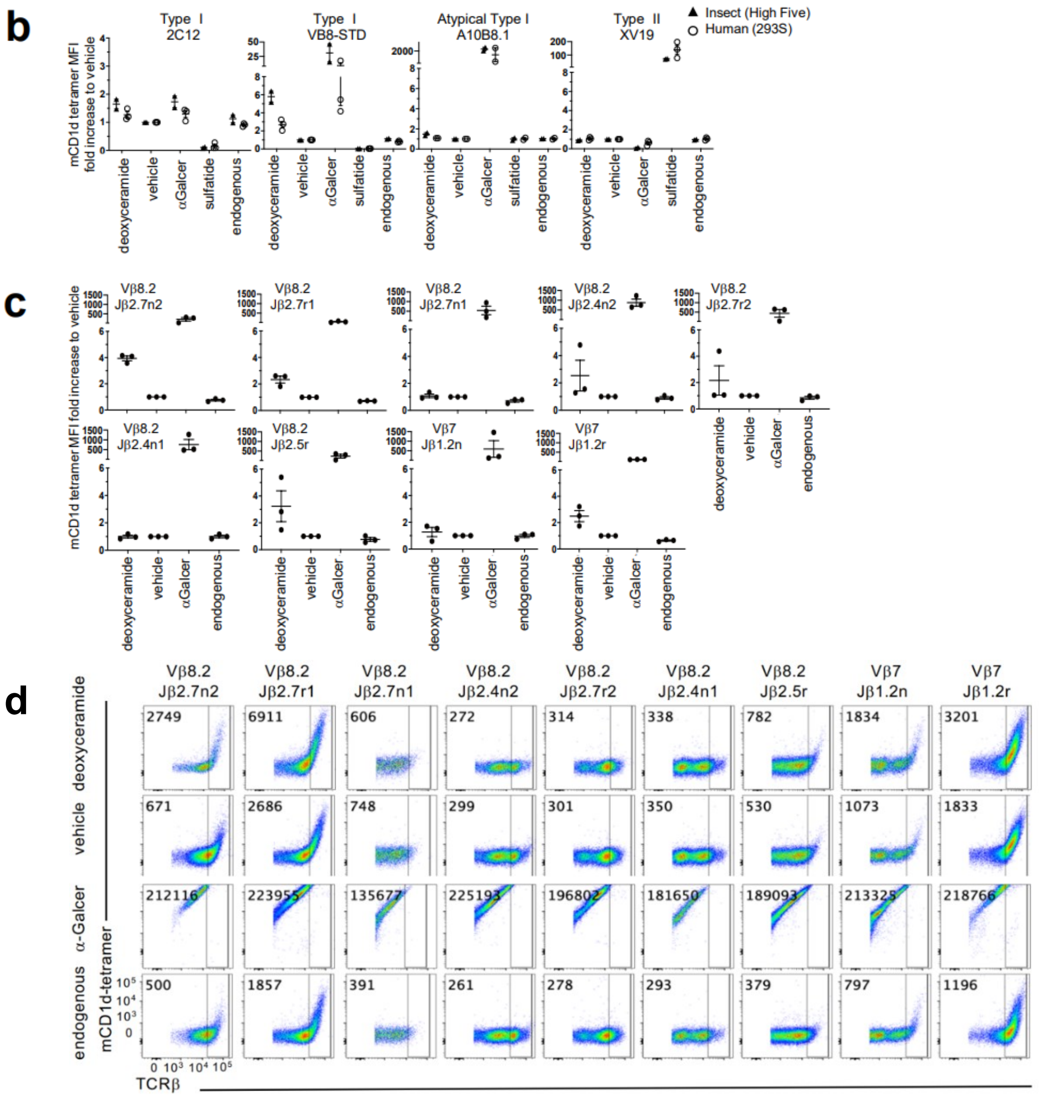

Fig. S6. Deoxyceramide recognition by murine NKT TCRs. a) Summary of NKT clones and variable (V) or joining (J) gene usage for TCRα or TCRβ chains, as per (1). The complementary determining region amino acid sequences are shown with residues partly or wholly encoded by non-germline insertions highlighted in bold. b) Mock or antigen treated mouse CD1d-tetramers were assessed for staining of BW58 lines expressing the indicated TCRs with cells gated for similar surface TCR expression +/- SEM from two experiments performed with mouse CD1d expressed in High Five cells and three experiments using proteins from 293S GNtI-/-. Plots from one representative experiment is shown in Figure 3f. c) Mouse CD1d-tetramers in 293S GNtI-/- cells were assessed for staining of type I NKT TCR-transduced BW58 lines expressing the TCRs indicated in (a). Graphs show MFI fold-increase compared to vehicle treated mCD1d-tetramers from three experiments performed, one representative shown in (d) Numbers in d represent mean fluorescence intensity (MFI) on cells with similar surface TCR expression. Each dot shown in graphs for (b) and (c) represents an independent experiment .

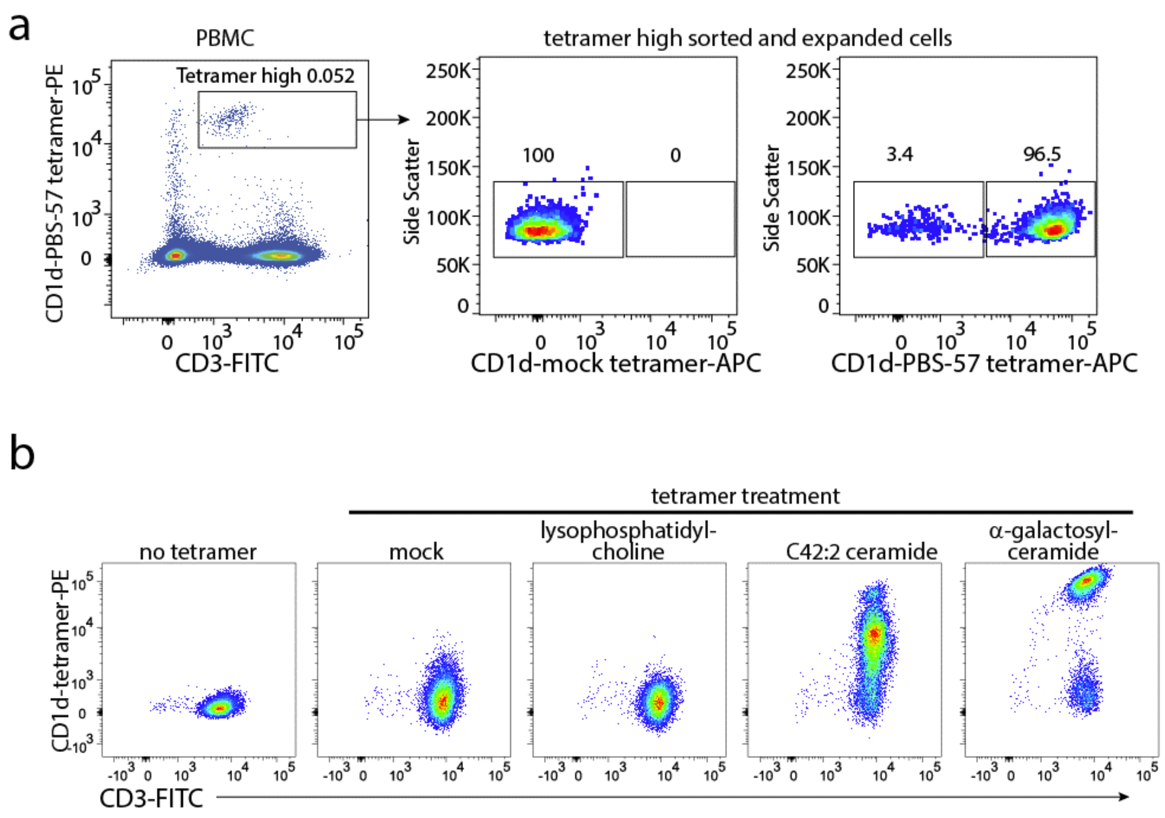

Fig. S7. Creation and tetramer staining of a human NKT cell-enriched cell line. a) CD1d-PBS-57 tetramer high cells were sorted from PBMC from a healthy donor and expanded. After the first expansion, the sorted cells were analyzed by flow cytometry, expanded again, yielding a line that is 96.5 percent Type I NKT cells, which was used for further experiments. b) Single tetramer stainings with a series of tetramers that were antigen or mock treated in-house in parallel.

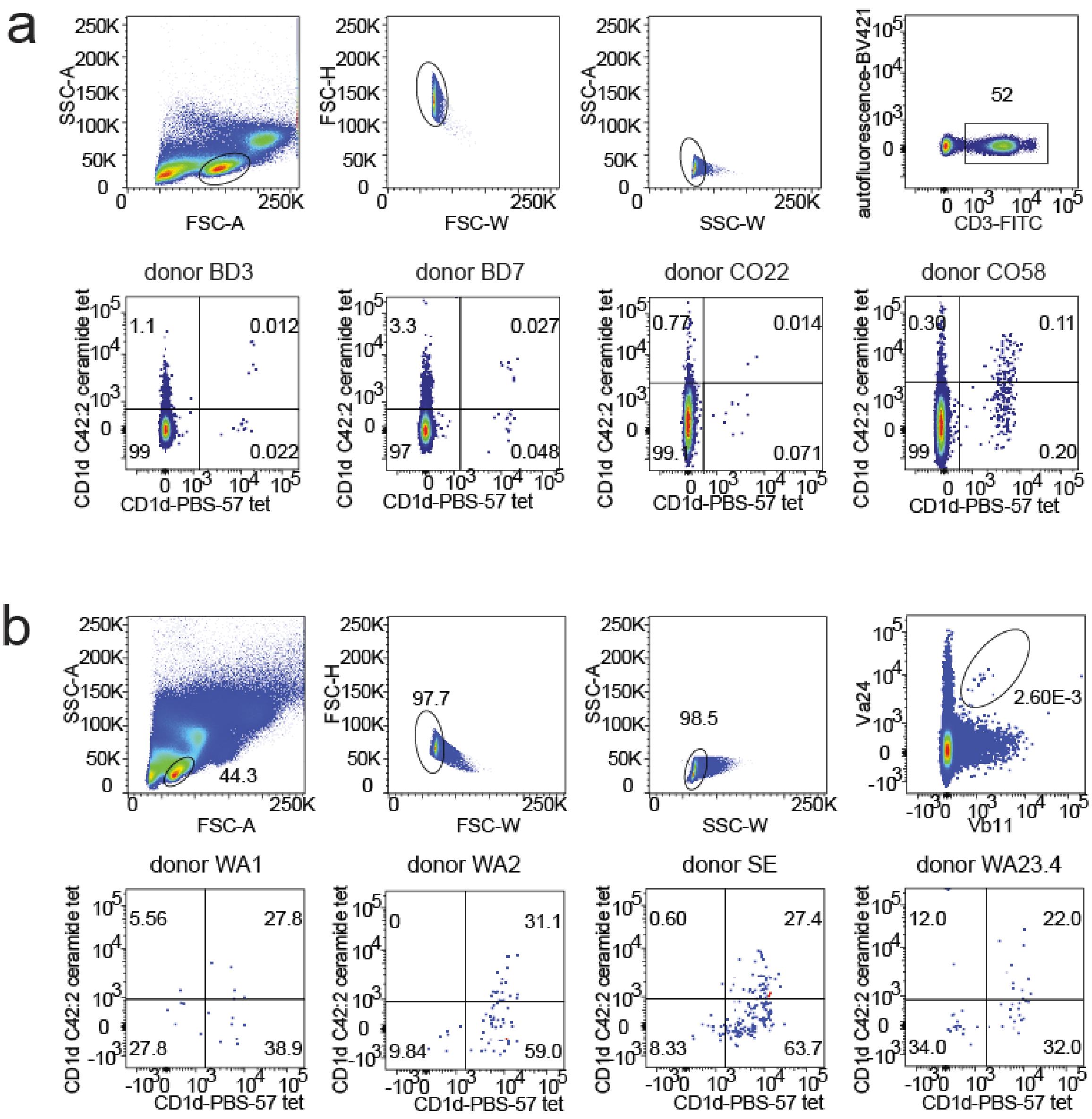

Fig. S8. CD1d tetramer staining of human PBMC. a) PBMCs of four donors were stained with anti-CD3 and CD1d-PBS57-APC and CD1d-ceramide 42:2-PE tetramers. After gating for CD3+ single lymphocytes (top row), CD1d tetramer binding was determined (lower row). b) PBMCs isolated from an additional four donors were stained with anti-Va24 and -Vb11 antibodies and CD1d-PBS57-APC and CD1d-ceramide 42:2-PE tetramers. CD1d-tetramer binding was assessed within Va24 and Vb11 double positive single lymphocytes. This gating strategy forms the basis for the data shown in Figure 3h.

Table S1. Data collection and refinement statistics

Data collection	2C12 TCR-mCD1d-Ceramide
Temperature	100k
Resolution limits (Å)	48.47-2.7 (2.8-2.7)
Space Group	$P2_12_12_1$
Cell dimensions (Å)	a=57.9, b=79.0, c=242.4 α=β=γ=90°
Total N°· observations	232520 (28178)
N°· unique observations	31771 (4050)
Multiplicity	7.3 (7.0)
Data completeness (%)	99.6 (97.2)
Wilson B-factors ($Å^2$)	61.5
I/σ_I	12.5 (2.4)
$R_{p.i.m}$ [1] (%)	4 (33.2)
**Refinement statistics**	
Rfactor[2] (%)	19.2
Rfree[3] (%)	26.5
Non hydrogen atoms	
- Protein	6649
- Water	26
- Heterogen	152
Ramachandran plot (%)	
- Most favored	95.23
- Allowed	4.65
- Disallowed	0.12
rmsd bonds (Å)	0.010
rmsd angles (°)	1.172

[1] $\mathbf{R_{p.i.m}} = \Sigma_{hkl} [1/(N-1)]^{1/2} \Sigma i \mid I_{hkl}, i - \langle I_{hkl} \rangle \mid / \Sigma_{hkl} \langle I_{hkl} \rangle$

[2] $\mathbf{R_{factor}} = (\Sigma \mid |Fo| - |Fc| \mid) / (\Sigma |Fo|)$ - for all data except as indicated in footnote 3.

[3] 5% of data was used for the R_{free} calculation.

Values in the parentheses refer to the highest resolution shell.

Table S2. 2C12 TCR contacts with ceramide and mCD1d.

TCR gene	TCR residues	CD1d residues	Bond type
CDR3α	Asp94-Oδ1	Arg79-Nη1	SB
CDR3α	Asp94-Oδ2	Arg79-Nη2	SB
CDR3α	Asp94	Arg79	VDW
CDR3α	Arg95-Nε	Asp80-Oδ1	SB
CDR3α	Arg95-Nη1	Ser76-O	HB
CDR3α	Arg95	Ser76, Arg79, Asp80	VDW
CDR3α	Gly96-N	Asp153-Oδ2	HB
CDR3α	Gly96-O	Ala152-O, Asp153-N	HB
CDR3α	Gly96	Ala152, Asp153	VDW
CDR3α	Ser97	Val149, Ala152	VDW
CDR3α	Leu99-O	Arg79-Nη1	HB
CDR3α	Leu99	Arg79, Asp80, Glu83, Met87, Val149	VDW
CDR3α	Gly100	Arg79	VDW
CDR3α	Arg103	Arg79, Glu83	VDW
CDR2β	Tyr48-Oη	Glu83-Oε2, Lys86-Nζ	HB
CDR2β	Tyr48	Glu83, Lys86	VDW
CDR2β	Tyr50-Oη	Glu83-Oε2	HB
CDR2β	Tyr50	Glu83, Lys86, Met87	VDW
CDR2β	Glu56	Lys86	VDW
CDR3β	Glu97	Ala152	VDW
**TCR gene**	**TCR residues**	**Ceramide atoms**	**Bond type**
CDR1α	Pro28	1-OH	VDW
CDR3α	Arg95	3-OH	VDW

HB: Hydrogen bond, VDW: van der Waals, Cut-off at 4 Å for VDW interactions and 3.5 Å for HB.

Considering the interacting OH groups in ceramide, the 1-OH group is missing from deoxyceramide, and both 1-OH and 3-OH are missing from diacylglycerol.

SI References

1. Cameron G*, et al.* (2015) Antigen Specificity of Type I NKT Cells Is Governed by TCR beta-Chain Diversity. *J. Immunol.* 195(10):4604-4614.
2. Matsuda JL*, et al.* (2000) Tracking the response of natural killer T cells to a glycolipid antigen using CD1d tetramers. *J. Exp. Med.* 192(5):741-754.
3. Almeida CF*, et al.* (2019) Distinct CD1d docking strategies exhibited by diverse Type II NKT cell receptors. *Nat Commun* 10(1):5242.
4. Cossarizza A*, et al.* (2021) Guidelines for the use of flow cytometry and cell sorting in immunological studies (third edition). *Eur J Immunol* 51(12):2708-3145.
5. Pellicci DG*, et al.* (2009) Differential recognition of CD1d-alpha-galactosyl ceramide by the V beta 8.2 and V beta 7 semi-invariant NKT T cell receptors. *Immunity* 31(1):47-59.
6. Garboczi DN*, et al.* (1996) Assembly, specific binding, and crystallization of a human TCR-alphabeta with an antigenic Tax peptide from human T lymphotropic virus type 1 and the class I MHC molecule HLA-A2. *J. Immunol.* 157(12):5403-5410.
7. Garboczi DN*, et al.* (1996) Structure of the complex between human T-cell receptor, viral peptide and HLA-A2. *Nature* 384(6605):134-141.
8. Birkinshaw RW*, et al.* (2015) alphabeta T cell antigen receptor recognition of CD1a presenting self lipid ligands. *Nat Immunol* 16(3):258-266.
9. Brennan PJ*, et al.* (2017) Structural determination of lipid antigens captured at the CD1d-T-cell receptor interface. *Proc Natl Acad Sci U S A* 114(31):8348-8353.
10. van 't Klooster JS*, et al.* (2020) Periprotein lipidomes of Saccharomyces cerevisiae provide a flexible environment for conformational changes of membrane proteins. *eLife* 9.
11. Govindarajan S*, et al.* (2018) Stabilization of cytokine mRNAs in iNKT cells requires the serine-threonine kinase IRE1alpha. *Nat Commun* 9(1):5340.
12. Jacques P*, et al.* (2010) Invariant natural killer T cells are natural regulators of murine spondylarthritis. *Arthritis Rheum.* 62(4):988-999.
13. Oh SF*, et al.* (2021) Host immunomodulatory lipids created by symbionts from dietary amino acids. *Nature* 600(7888):302-307.
14. Kabsch W (2010) Xds. *Acta Crystallogr. D Biol. Crystallogr.* 66(Pt 2):125-132.
15. Evans P (2006) Scaling and assessment of data quality. *Acta Crystallogr. D Biol. Crystallogr.* 62(Pt 1):72-82.
16. Adams PD*, et al.* (2010) PHENIX: a comprehensive Python-based system for macromolecular structure solution. *Acta Crystallogr. D Biol. Crystallogr.* 66(Pt 2):213-221.
